# A site-moiety map and virtual screening approach for discovery of novel 5-LOX inhibitors

**DOI:** 10.1038/s41598-020-67420-9

**Published:** 2020-06-29

**Authors:** Kai-Cheng Hsu, Wei-Chun HuangFu, Tony Eight Lin, Min-Wu Chao, Tzu-Ying Sung, Yi-Ying Chen, Shiow-Lin Pan, Jih-Chin Lee, Shey-Cherng Tzou, Chung-Ming Sun, Jinn-Moon Yang

**Affiliations:** 1 Graduate Institute of Cancer Biology and Drug Discovery, College of Medical Science and Technology, Taipei Medical University, Taipei, Taiwan; 2 Ph.D. Program for Cancer Molecular Biology and Drug Discovery, College of Medical Science and Technology, Taipei Medical University, Taipei, Taiwan; 3 Ph.D. Program in Biotechnology Research and Development, College of Pharmacy, Taipei Medical University, Taipei, Taiwan; 4 Biomedical Commercialization Center, Taipei Medical University, Taipei, Taiwan; 5 Institute of Bioinformatics and Systems Biology, National Chiao Tung University, Hsinchu, Taiwan; 6 Department of Otolaryngology-Head and Neck Surgery, Tri-Service General Hospital, Taipei, Taiwan; 7 Department of Otolaryngology-Head and Neck Surgery, National Defense Medical Center, Taipei, Taiwan; 8 Department of Biological Science and Technology, National Chiao Tung University, Hsinchu, Taiwan; 9 Department of Applied Chemistry, National Chiao Tung University, Hsinchu, Taiwan; 10 Center for Intelligent Drug Systems and Smart Bio-Devices, National Chiao Tung University, Hsinchu, Taiwan

**Keywords:** Small molecules, Drug discovery, Drug screening, Virtual screening

## Abstract

The immune system works in conjunction with inflammation. Excessive inflammation underlies various human diseases, such as asthma, diabetes and heart disease. Previous studies found that 5-lipoxygenase (5-LOX) plays a crucial role in metabolizing arachidonic acid into inflammatory mediators and is a potential therapeutic target. In this study, we performed an in silico approach to establish a site-moiety map (SiMMap) to screen for new 5-LOX inhibitors. The map is composed of several anchors that contain key residues, moiety preferences, and their interaction types (i.e., electrostatic (E), hydrogen-bonding (H), and van der Waals (V) interactions) within the catalytic site. In total, we identified one EH, one H, and five V anchors, within the 5-LOX catalytic site. Based on the SiMMap, three 5-LOX inhibitors (YS1, YS2, and YS3) were identified. An enzyme-based assay validated inhibitory activity of YS1, YS2, and YS3 against 5-LOX with an IC_50_ value of 2.7, 4.2, and 5.3 μM, respectively. All three inhibitors significantly decrease LPS-induced TNF-α and IL-6 production, which suggests its potential use an anti-inflammatory agent. In addition, the identified 5-LOX inhibitors contain a novel scaffold. The discovery of these inhibitors presents an opportunity for designing specific anti-inflammatory drugs.

## Introduction

Inflammation is an essential biological process in response to injury and infection. This process regulates blood flow and vascular permeability by releasing a variety of inflammatory mediators. This results in activation and migration of leukocytes into inflammatory sites. Inflammation and cancer are linked by specific oxidative processes in the tumor microenvironment^1^. Similarly, excessive or inappropriate inflammation may cause various diseases, such as asthma, diabetes, and heart disease^2–4^. Lipoxygenases are enzymes that can be found in both plants and animals and play a crucial role in the inflammatory process^5,6^. Humans contain six lipoxygenase isozymes that are named after their site of substrate oxygenation^6,7^. As a result, each lipoxygenase can target the same substrate, but produce a different product. As a result, lipoxygenases are potential therapeutic targets for various diseases.

Arachidonic acid (AA) is a key inflammatory intermediate. When leukocytes are activated, AA is released from the nuclear membrane by phospholipase A2^8,9^. Tumor necrosis factor-alpha (TNF-α) stimulates phospholipase A2 activity^10^. A lipoxygenase isozyme, 5-lipoxygenase (5-LOX) will then catalyze AA to produce pro-inflammatory cytokines, such as interleukin 6 (IL-6)^9^. Previous research has shown that overexpression of TNF-α and IL-6 can increase inflammation in cultured monocytes^11^. 5-LOX is overexpressed in colon, lung, prostate, and oral cancers^12–14^. As a result, several types of 5-LOX inhibitors have been developed for cancer treatments^15–17^. Currently, only one 5-LOX inhibitor, Zileuton, has been approved by the FDA for the treatment of asthma^18^. Studies have focused on expanding Zileuton’s use towards other inflammatory disease involving leukotrienes^19,20^. However, Zileuton efficacy is limited due to side effects (e.g., liver toxicity), short duration within the body, and large and frequent dosages due to poor pharmacokinetic profile hindering its use in many inflammatory diseases^21–23^. As a result, there is a need for new 5-LOX inhibitors that not only overcome these issues, but also effectively combat inflammatory associated diseases.

A site-moiety map (SiMMap) can be used to identify key pharmacophore features that trigger or block a biological response^24^. A SiMMap is comprised of several anchors. Each anchor contains the following elements: (1) a binding pocket with consensus interacting residues; (2) moiety composition of the binding pocket; and (3) the type of interaction between protein and ligand^24^, including electrostatic (E), hydrogen-bonding (H), and van der Waals (V) interactions. Anchors are derived by exploiting thousands of docked protein–compound complexes. A compound that matches more anchors has higher potential for interacting with the targeted site. SiMMaps offers several advantages to enhance virtual screening accuracy and has been successfully applied to identify inhibitors targeting shikimate kinase, tyrosine kinase and neuraminidase^25–27^. In addition, moiety preferences of anchors provide guidelines for lead optimization. In this study, we aimed to identify novel 5-LOX inhibitors. We first established a 5-LOX SiMMap. Compounds were filtered based on their SiMMap score. We identified potential 5-LOX inhibitors with a maleimide scaffold. Cellular assays identified three inhibitors that suppress 5-LOX activity and reduce expression levels of pro-inflammatory mediators TNF-α and IL-6^9^. Therefore, the identification of 5-LOX inhibitors with novel scaffolds by establishing a SiMMap of the 5-LOX catalytic site presents a new prospect for designing novel anti-inflammatory drugs.

## Results

### Overview of discovering new 5-LOX inhibitors

**Figure 1:**
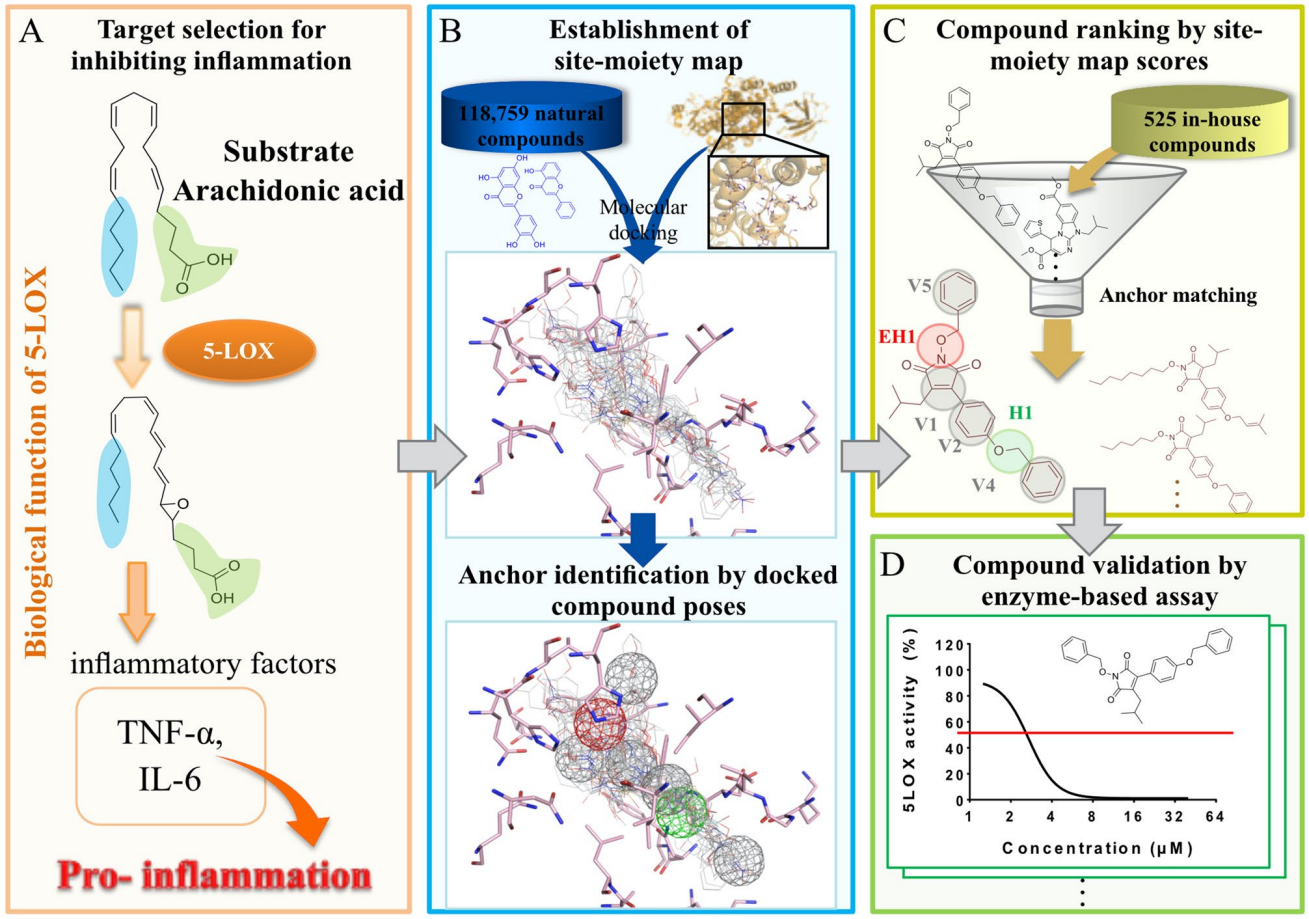
Overview of establishing a SiMMap and virtual screening. (**A**) 5-LOX metabolizes arachidonic acid to produce various inflammatory factors. (**B**) A SiMMap was established and anchor residues were identified for their moiety preference. (**C**) 525 in-house compounds were docked and screened based on their SiMMap ranking. (**D**) High ranked compounds were selected for enzyme-based assay validation.

Figure 1 shows the major steps for establishing a 5-LOX SiMMap and the virtual screening workflow. 5-LOX plays an important role in synthesizing leukotrienes, which are known inflammatory mediators, from arachidonic acid^21^. However, excessive inflammation can lead to various diseases, making 5-LOX an attractive target for potential cancer treatment^12–14^. Inhibition of 5-LOX can potentially block production of pro-inflammatory factors such as TNF-α or IL-6 (Fig. 1A). First, we modeled the structure of 5-LOX due to missing residues in the catalytic site of the open-form crystal structure. The 5-LOX contains a “cork” structural motif that facilitates access to the catalytic site^28^. Next, 118,759 natural compounds collected from the ZINC compound database^29^ were then docked into the 5-LOX catalytic site using the molecular docking tool GEMDOCK^30,31^. The compounds were ranked by their docking score and the top 2,000 compounds were used to construct the SiMMap of 5-LOX (Fig. 1B). The SiMMap consists of anchors that are identified within the catalytic site^24^. An anchor is derived by identifying a catalytic site pocket with consensus interactions between residues and moieties. Neighboring interacting residues have a specific interaction type with compound moieties. Finally, 525 in-house synthesized compounds were docked and then ranked based on their SiMMap score (Fig. 1C). Compounds with high scores were considered potential 5-LOX inhibitors and were validated by enzyme-based assay (Fig. 1D).

### Site-moiety map of 5-LOX

**Figure 2:**
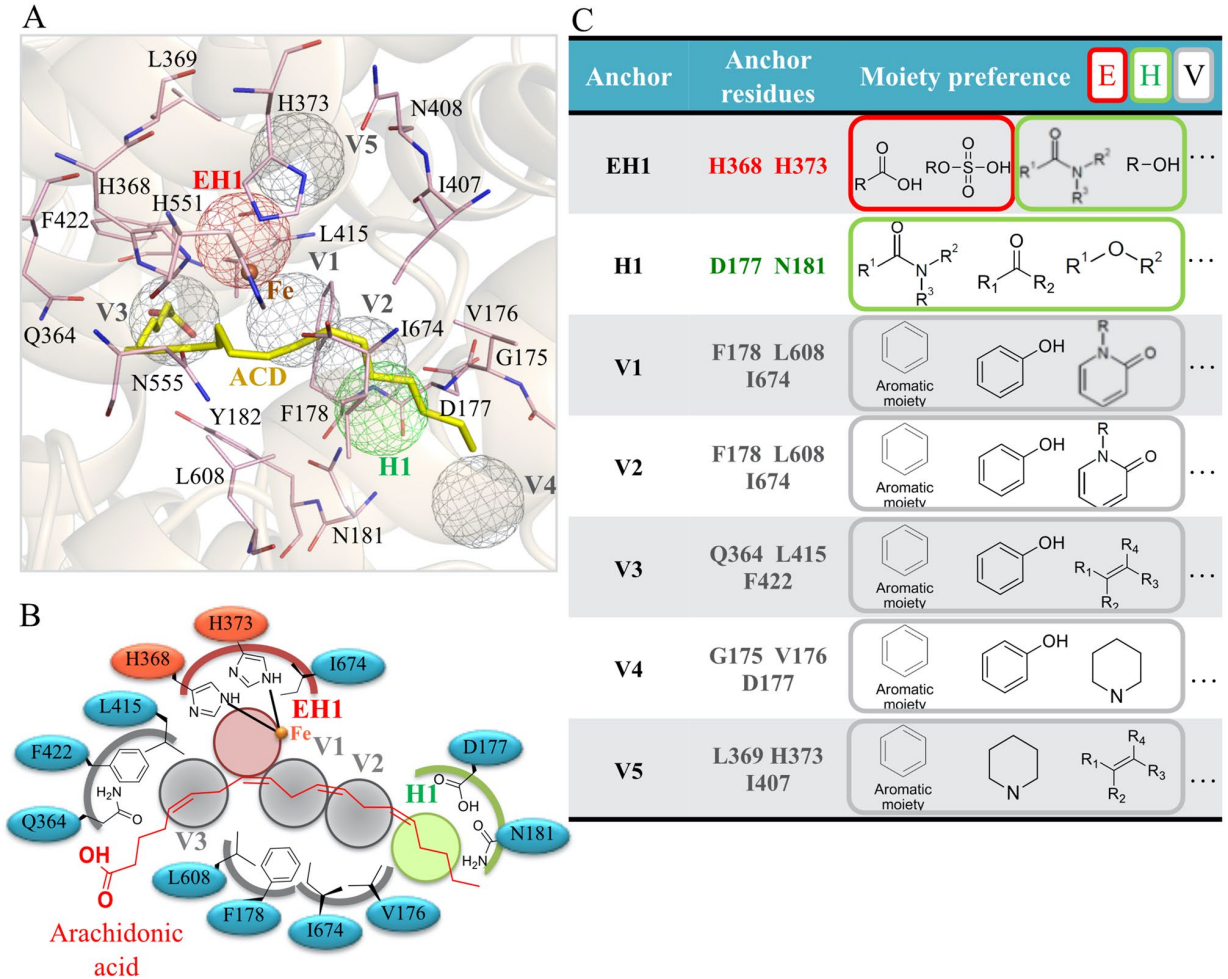
SiMMap of 5-LOX. (**A**) Arachidonic acid (yellow) is docked in the 5-LOX catalytic site (pink). The anchors are represented as electrostatic (red), hydrogen-bonding (green), or van der Waals (gray) (**B**) A 2D map of the 5-LOX catalytic site with arachidonic acid (red). Interacting anchors and catalytic site residues are labeled as shown. (**C**) The anchors have an interaction type and moiety preference.

The SiMMap of 5-LOX consists of seven anchors: one (EH1), one hydrogen (H1), and five van der Waal (V1 − V5) (Fig. 2A). Anchor EH1 is located adjacent to the metal ion (Fe^2+^) and consists of two positively-charged residues: H368 and H373 (Fig. 2B). The EH1 anchor favors polar moieties that form both electrostatic and hydrogen-bonding interactions (Fig. 2C). Two residues (D177 and N181) of the H1 anchor form a polar binding pocket. The H1 anchor has a preference for hydrogen bonds with amide, ketone, and polar moieties. Interestingly, the substrate, arachidonic acid, does not yield a hydrogen bond with the H1 anchor residues, which suggests that inhibitors with hydrogen-bonding interactions could have favorable binding affinity towards 5-LOX (Fig. 2B).

There are five van der Waals anchors in the catalytic site of 5-LOX, which indicate that the catalytic site is hydrophobic and may have a preference for inhibitors with large aromatic moieties (Fig. 2). The V1 anchor is situated near the EH1 anchor and consists of three hydrophobic residues: F178, L608, and I674. These three residues form a hydrophobic channel in the catalytic site and frequently yield stable van der Waals interactions with ring groups of docked compounds, such as aromatic, phenol, and heterocyclic groups. Residue F178, along with the aromatic residue Y182, forms a “cork” that plays a critical role in allowing access to the catalytic site^28^. The V2 anchor includes residues V176, F178, and L608. While the V2 anchor is in a different location, it shares residues that make up the V1 anchor. The long side chains of the residues favor van der Waals interactions with docked compounds. Three residues (Q364, L415, and F422) with long side chains comprise the V3 anchor. The frequently interacting moieties of this anchor are aromatic rings, phenols, and alkenes. Arachidonic acid makes van der Waals contacts with these three anchors sites (Fig. 2B), further suggesting the importance of the anchors for substrate binding.

Anchors V4 and V were not observed in the binding region of arachidonic acid (Fig. 2A, B). The V4 anchor consists of residues G175, V176, and D177. Anchor V5 is comprised of residues L369, H373, and N407. Both anchors prefer van der Waals interactions with aromatic rings, heterocyclic groups, and alkene. While these anchors do not directly interact with arachidonic acid, they provide additional regions for forming compound interactions. This suggests compounds with interactions at anchors V4 and V5 may yield 5-LOX inhibitors with novel structures.

We further validated our docking results and SiMMap by docking 990 randomly selected compounds from the Advanced Chemical Directory (ACD) and 10 known 5-LOX inhibitors obtained from BindingDB compound database^32^. The IC_50_ values of the 10 active compounds are less than 1 µM (Supplementary Fig. 1). These known compounds were then ranked based on their SiMMap score. Overall, the known 5-LOX inhibitors were ranked higher based on their SiMMap score compared to their docking score alone (Supplementary Fig. 2). This suggests that the SiMMap scoring can help increase identification of potential inhibitors.

### Evolutionary conservation of 5-LOX anchors

**Figure 3:**
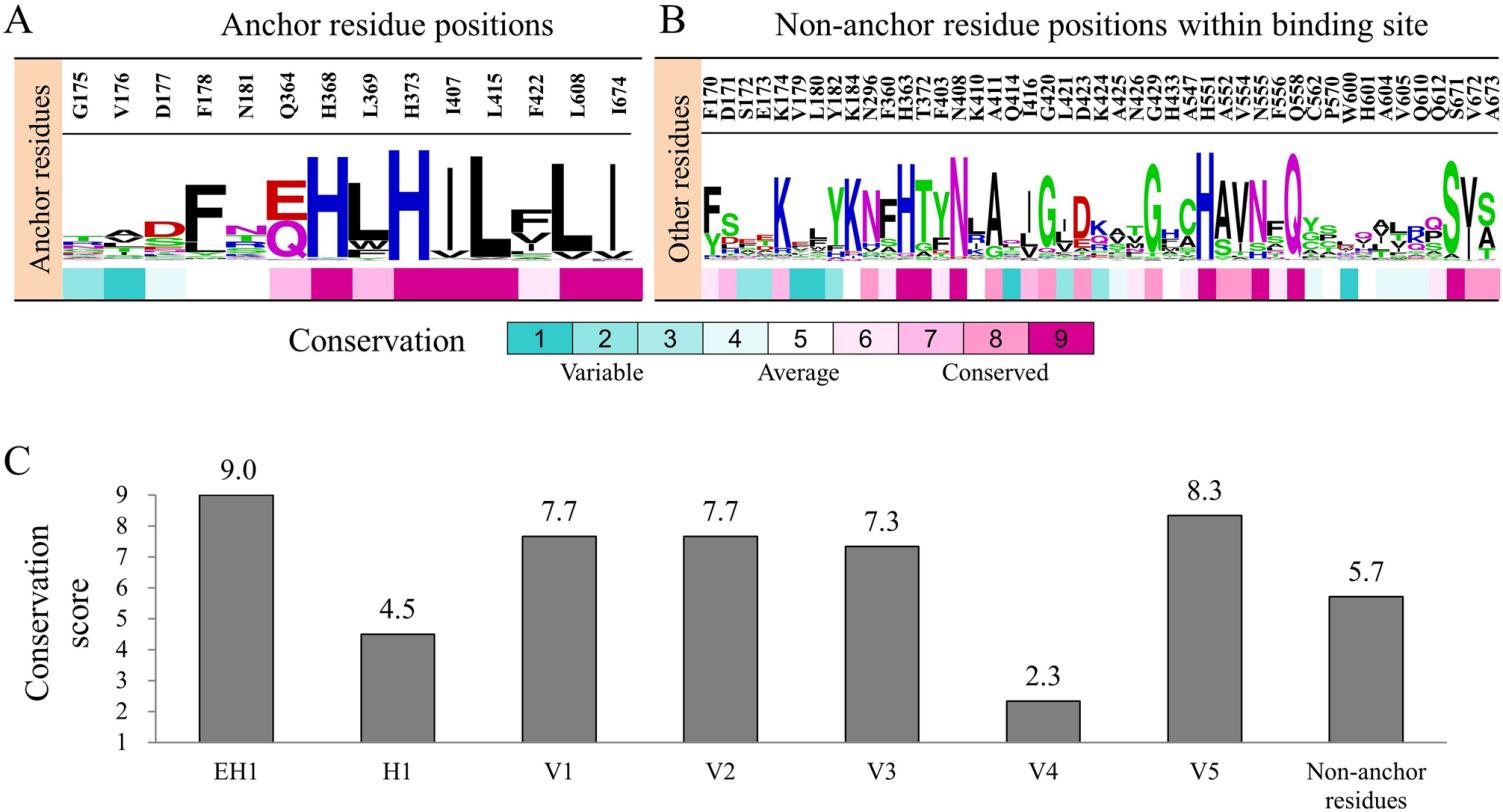
Comparison of conservation scores. Conservation-score of (**A**) anchor residues and (**B**) non-anchor residues in the catalytic site is scored as 1 (least conserved) to 9 (most conserved). (**C**) The average conservation-score of anchor residues and non-anchor residues.

The Lipoxygenase family retain conserved amino acids at the catalytic center^5^. To determine the importance of the SiMMap anchors through an evolutionary view, we examined the evolutionary conservation of 5-LOX catalytic site residues (Fig. 3A, B). A conservation score for each residue position was obtained from the ConSurf server^33^. The server generated a multiple sequence alignment of the 5-LOX homologous sequences and used the alignment to measure the conservation degree of each position. Conservation scores ranged from 1 to 9, which indicates the least conserved and the highest conserved positions, respectively. The catalytic site residues were divided into anchor and non-anchor residues (Fig. 3A, B). A conservation score for each anchor was calculated by averaging the conservation score of its anchor residues. Overall, anchor residues are more conserved than the non-anchor residues with the exception of anchors H1 and V4 (Fig. 3C).

The EH1 anchor is the most conserved due to residues H368 and H373 having an essential role in coordinating to the metal ion^8^. Mutations with these residues result in the loss of 5-LOX activity^34^. The V1, V2, and V3 anchors are slightly less conserved; their conservation score is 7.7, 7.7, and 7.3, respectively (Fig. 3C). Residues F178, L415, and L608 are hydrophobic residues that constitute anchors V1-3. Their conserved nature further confirms their importance for inhibiting 5-LOX. Both anchors V4 and V5 are located outside of the traditional 5-LOX catalytic site and are not involved in the metabolism of arachidonic acid (Fig. 2A). However, anchor V4 is the least conserved anchor with a conservation score of 2.3 (Fig. 3A). The V4 anchor contains three non-conserved residues, G175, V176, and D177 whose score is 1, 2, and 5 respectively. In contrast, anchor V5 was found to be the second most conserved anchor. The conservation scores of the V5 anchor residues L369, H373, and I407 are 7, 9, and 9, respectively. Together, this suggests that compound interactions with the V4 and V5 anchor may lead to novel 5-LOX inhibitors.

### Identification of novel 5-LOX inhibitors

**Figure 4:**
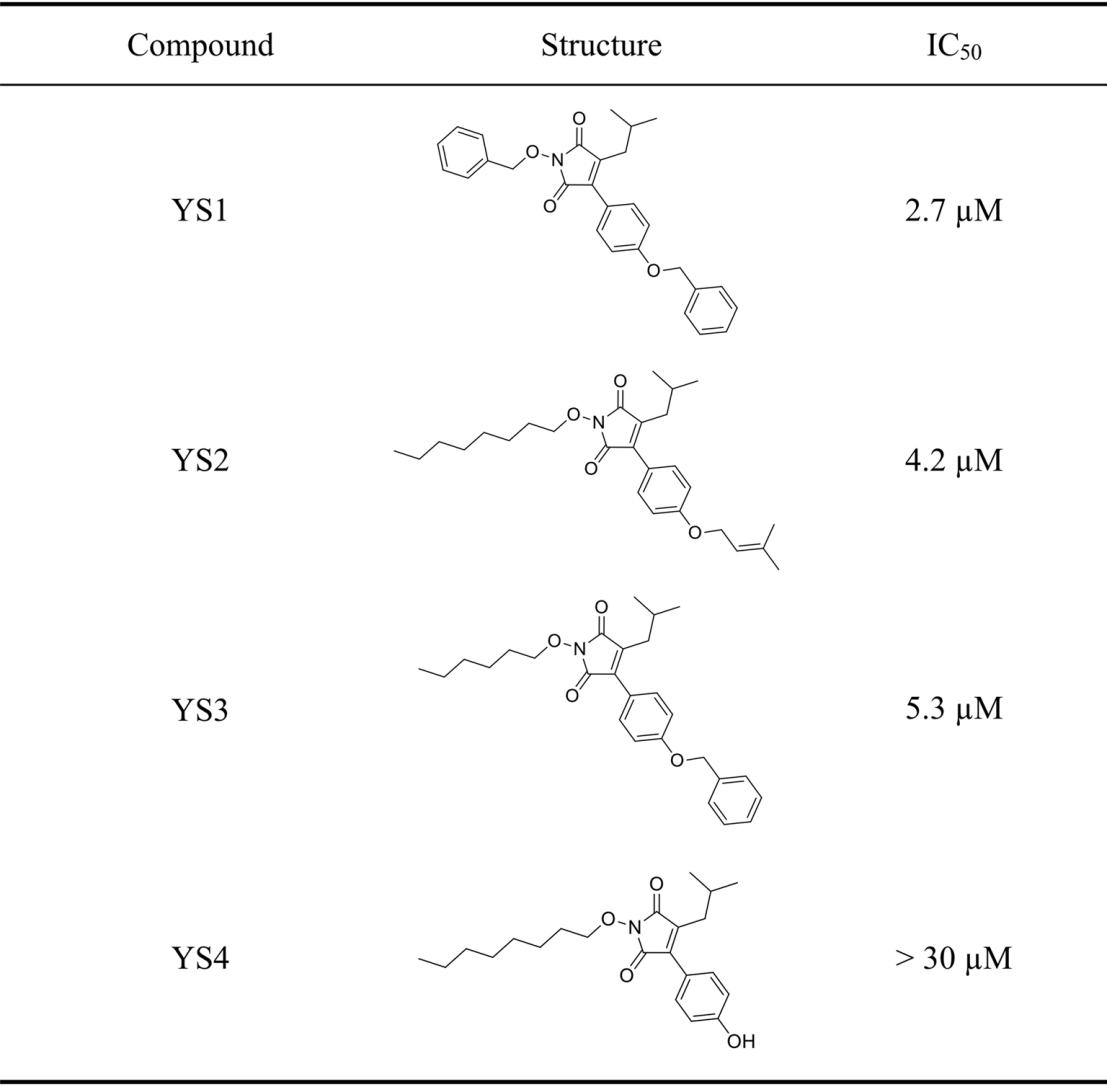
Identified compounds have dose dependent IC_50_ values. The IC_50_ values for compound YS1, YS2, YS3, and YS4 against 5-LOX was obtained. Compound YS4 did not show sufficient inhibition.

We docked 525 in-house, synthesized compound library into the catalytic site of 5-LOX and ranked them based on their SiMMap score. The top 120 ranked compounds can be found in Table S1. Four compounds were selected to evaluate their activities against 5-LOX. The top ranked compounds included YS1, YS2 and YS3. These compounds also form hydrogen bonds with residues H368 and H373 of the EH1 anchor. It has been suggested that residue H368 and H373 play an essential role in coordinating to the metal ion^8^. Both residues are highly conserved (Fig. 3A). We have also examined the structures of the top 120 compounds in this study. Compound YS4, which was ranked 14 from the SiMMap, has the same scaffold as the top 3 compounds; thus, it was also selected for validation. Of the four, compounds YS1, YS2, and YS3 inhibit 5-LOX with IC_50_ values of 2.7, 4.2, and 5.3 μM, respectively. In contrast, the compound YS4 produced a larger IC_50_ and did not show effective 5-LOX inhibition (Fig. 4).

**Figure 5:**
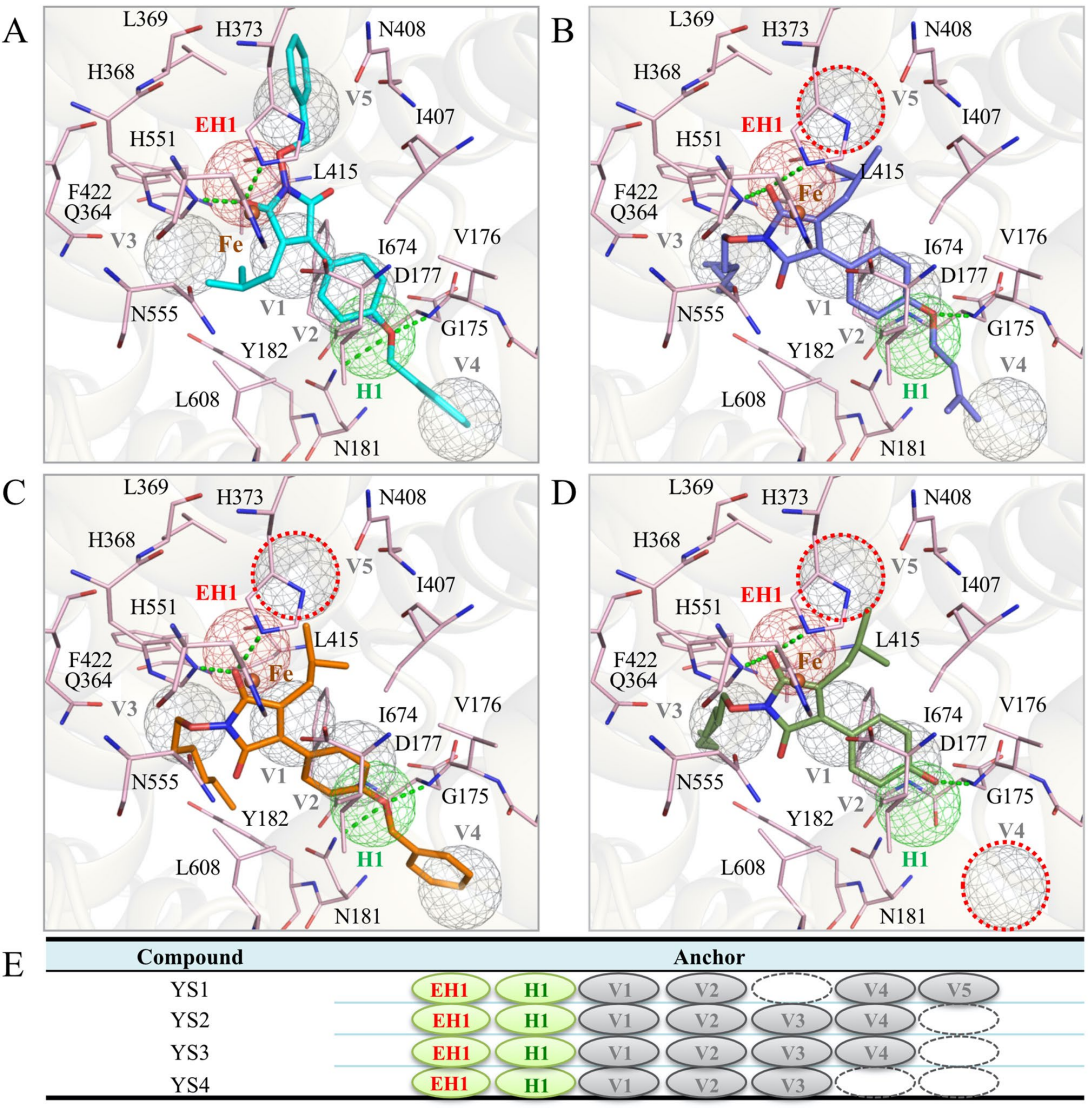
Structure–activity relationship of identified inhibitors and 5-LOX. The catalytic site of 5-LOX (pink) is shown with the docking pose of compounds YS1 (**A**, blue), YS2 (**B**, purple), YS3 (**C**, orange), and YS4 (**D**, green). The anchor spheres are highlighted and colored as seen in Fig. 2. Residues and anchors are labeled as shown. Green lines denote hydrogen bonds. Anchors with no interactions are highlighted in dotted red lines. (**E**) A table denoting interactions between the designated compound and the 5-LOX anchor sites.

Next, we performed an interaction analysis to determine the compound interactions within the 5-LOX catalytic site. YS1 was identified to be the most potent inhibitor against 5-LOX and produced the lowest IC_50_ value at 2.7 µM (Fig. 4). The docked pose of YS1 has one of its ether groups located within the H1 anchor and formed two hydrogen bonds with residues D177 and N181 (Fig. 5A). The maleimide scaffold of YS1 is sandwiched by residues of the V1 anchor (F178, L608, and I674), which facilitates van der Waals interactions. Although YS1 does not contain a negatively-charged functional group to form electrostatic interactions with the EH1 anchor residues, one of the ketone group on the maleimide ring yields two hydrogen-bond interactions with EH1 anchor residues H368 and H373. YS1 contains three benzene rings, one of which is located in the V2 anchor. This ring creates van der Waals interactions with the long side chains of the anchor residues. The interactions are similar to those formed by the carbon chain of arachidonic acid. The remaining two benzene rings occupy the catalytic pockets of the V4 and V5 anchors and have van der Waals contacts with the anchor residues. As mentioned previously, arachidonic acid does not bind to anchors V4 and V5. YS1 forms interactions with these anchors. This suggests that an inhibitor interaction with V4 and V5 can lead to a 5-LOX inhibitor.

YS2 and YS3 have similar activities and binding conformations (Fig. 5B, C). The benzene ring that branches from the nitrogen in YS1 is replaced with a long carbon-chain in compounds YS2 and YS3. The absence of a benzene ring decreases van der Waals interactions with the V5 anchor residues. However, YS2 and YS3 make additional van der Waals contacts with the V4 anchor; this additional interaction may account for the similar inhibitory effects of the three compounds. Both YS2 and YS3 possess a long carbon-chain functional group that forms van der Waals interactions with the V4 anchor residues G175 and V176. In comparison, YS4 lacks a large functional group that form interactions with the V4 and V5 anchor residues (Fig. 5D, E), which results in the inactivity of the compound against 5-LOX. Thus, the interactions with the V4 and V5 anchors may explain the favorable IC_50_ values of YS1, YS2 and YS3.

A molecular dynamic (MD) simulation was performed to identify the frequency of interactions between the residues and the identified inhibitors. Results of the MD simulation showed that the inhibitors continue to bind to the 5-LOX catalytic site after 10 ns (Supplementary Fig. 3A–C). Hydrogen-bond interactions were observed with all three inhibitors and occurred at a high frequency with residues D177 and H368, which form part of the H1 and EH1 anchor, respectively (Supplementary Fig. 3D). This suggests that hydrogen bonds formed between these residues and the identified inhibitors were necessary for 5-LOX inhibition.

The three inhibitors exhibited different hydrophobic interaction frequencies. Compound YS1 contains several benzene rings that facilitate hydrophobic interactions. A benzene moiety forms hydrophobic interactions with residues F178, L608 and I674, which constitute the V2 anchor. Another benzene moiety occupies a hydrophobic pocket and forms interactions to residues L369, I416 and R412. This region aligns closely to the V5 anchor (Fig. 5). Compound YS2 forms hydrophobic interactions with residues L180 and I407. Interactions with these residues were not observed with the other identified inhibitors. The eight-carbon chain of compound YS2 extends into a pocket to form hydrophobic interactions with residues F422, A604, Y182 and A425 (Supplementary Fig. 3D). In contrast, compound YS3 contains a six-carbon chain that extends into the same aforementioned pocket, but does not form as many hydrophobic interactions (Supplementary Fig. 3D). The carbon chain length and the difference in hydrophobic interactions may explain the different potencies observed between compounds YS2 and YS3 (Fig. 4). Overall, the MD simulation showed that the compounds have favorable interactions to the 5-LOX catalytic residues.

### Effects of the compounds on TNF-α and IL-6 production

**Figure 6:**
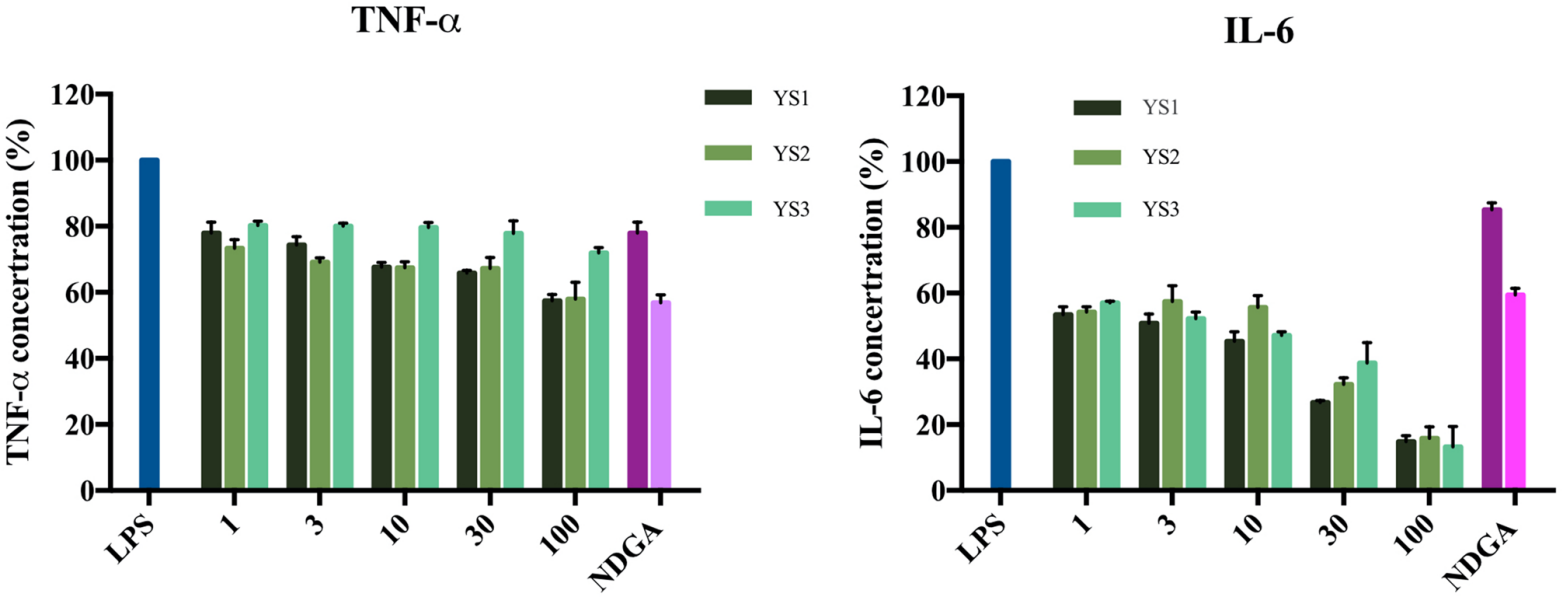
Identified compounds reduce pro-inflammatory cytokines. The RAW 264.7 cells were treated with YS1, YS2 and YS3 at concentrations of 1, 3, 10, 30 and 100 µM, respectively. IL-6 and TNF-α concentration is reduced in a dose dependent manner.

It has been reported that inhibition of 5-LOX attenuates inflammation in lipopolysaccharide (LPS) induced murine models^35^. Therefore, we evaluated whether the identified inhibitors would have an effect on LPS-induced pro-inflammatory cytokine release in vitro*.* TNF-α and IL-6 play an important role in mediating inflammation. In addition, 5-LOX inhibitors can ameliorate the pro-inflammatory mediators TNF-α and IL-6^9^. The three 5-LOX inhibitors exhibited a concentration-dependent reduction of TNF-α and IL-6 production in RAW.264 macrophages (Fig. 6). The identified inhibitors also significantly reduced TNF-α and IL-6 when compared to nordihydroguaiaretic acid (NDGA). As a result, the identified inhibitors reduce the expression of pro-inflammatory mediators TNF-α and IL-6 and have potential for use as an anti-inflammatory agent.

### Identified 5-LOX inhibitors are novel structures

**Figure 7:**
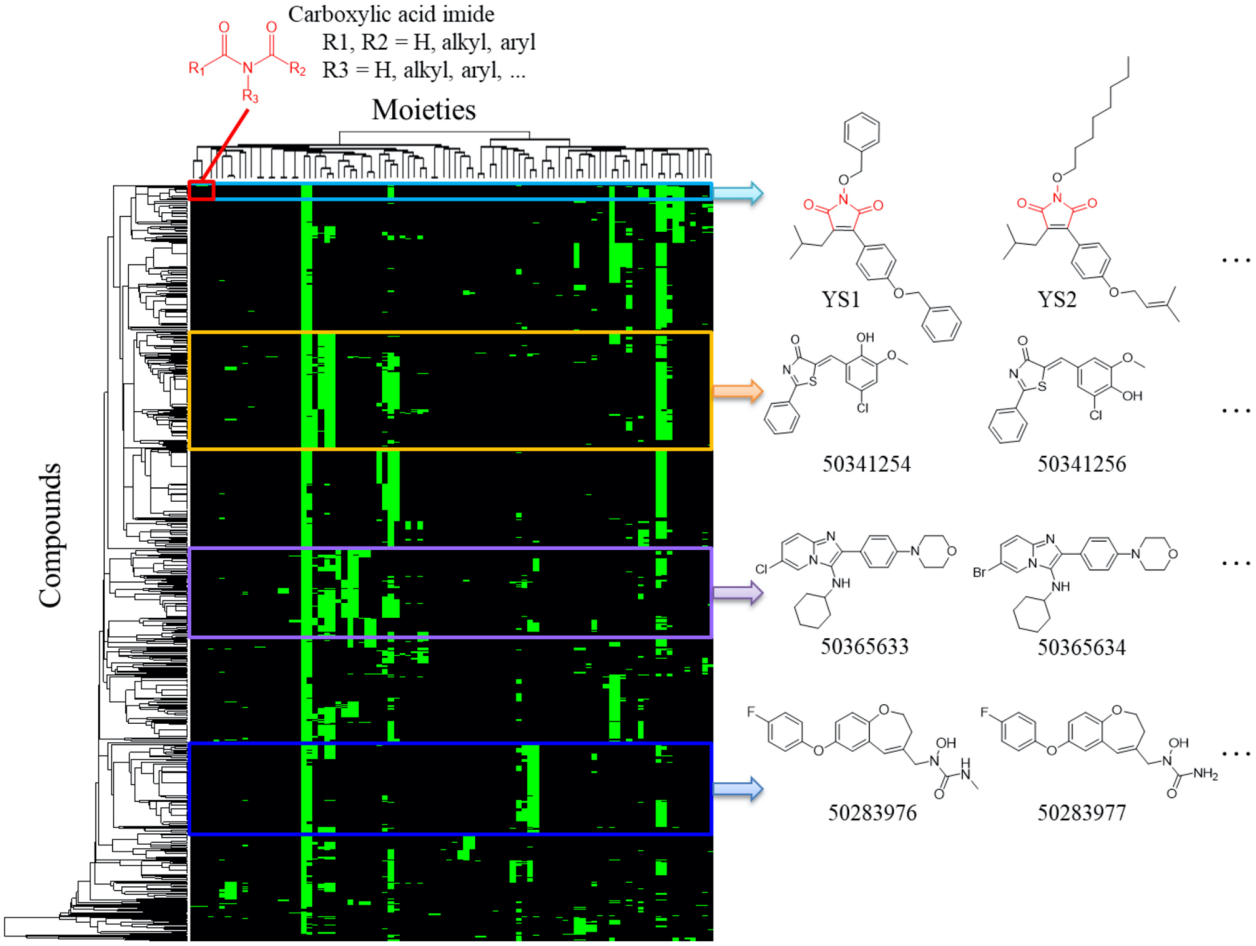
Interaction heatmap reveals compound moieties. Known 5-LOX inhibitors were grouped according to their moieties. Each colored box corresponds to a structural analog family. Representative analogs for each group are shown.

To assess the novelty of the identified 5-LOX inhibitors, we compared the three new 5-LOX inhibitors with other known inhibitors collected from the BindingDB database^32^ (Fig. 7). The analysis of moiety compositions shows that YS1, YS2, and YS3 were not clustered with other inhibitors. This suggests that the three compounds are 5-LOX inhibitors with a novel structure. The compounds contain a maleimide scaffold. The oxygen atom of the scaffold can form hydrogen-bonding interactions with the two metal-coordinating residues, H368 and H373 (Fig. 7). The inhibitors in this study add structural variety for 5-LOX inhibitor research. Our results suggest that these compounds can be used as lead compounds for developing a 5-LOX inhibitor.

## Discussion

There have been studies focusing on expanding the FDA approved inhibitor Zileuton’s use in inflammatory diseases^19,20^. However, Zileuton exhibits poor pharmacokinetic profile and half-life^21–23^. A novel 5-LOX inhibitor is needed to overcome these issues. In the present study, we sought to identify potential 5-LOX inhibitors using an in silico method. A 5-LOX SiMMap was first established. A SiMMap can infer physicochemical properties of a protein catalytic site and facilitate identification of moiety preferences to aid in the discovery of new inhibitors^36^. Next, we docked our in-house compound library using GEMDOCK and then filtered compounds based on their SiMMap score. The library consists of derivatives of potential inhibitors that have shown anti-inflammatory or anti-cancer effects. The top ranked compounds, YS1, YS2 and YS3, were selected for analysis (Table S1). Their structures are derivatives of Antrodin C, which is found in a mushroom commonly used in traditional Chinese medicine for treating a variety of ailments, such as skin irritation or inflammation^37^.

The SiMMap has been used to identify novel small molecule inhibitors targeting the shikimate kinase or neuraminidase^25,26^. To our knowledge, this study was the first to create a SiMMap for the 5-LOX catalytic site. We identified various anchors in the 5-LOX catalytic site that revealed interacting residues and specific physiochemical properties that influence ligand interactions (Fig. 2). The anchors consists of pockets within the catalytic site that contain interacting consensus residues grouped by physio-chemical properties. As a result, the anchors provide potential leads for optimal steric, hydrogen bonding or electrostatic moieties^24^. The SiMMap was validated by docking 990 randomly selected compounds from ACD along with 10 known 5-LOX inhibitors. The 5-LOX inhibitors ranked higher based on their SiMMap score (Supplementary Fig. 2). Altogether, the SiMMap provides useful information to identify potential inhibitors.

The LOX family contains a U-shape catalytic site that accounts for the large diversity of products^7^. This cavity is protected by a structural motif, sometimes described as a “cork”^8,28^. The obstruction of the “cork” residue F178 leads to a significant loss of enzyme function^28^. Evolutionary conserved residues can give insight into important interactions for 5-LOX function. Our SiMMap identified residue F178 in anchors V1 and V2, which aligns with the traditional binding region of arachidonic acid in the 5-LOX catalytic site (Fig. 2A, B). Residue F178 is highly conserved (Fig. 3). Residues H368 and H373 form part of the EH1 anchor and play a key role in coordinating to the 5-LOX metal ion^8^. The SiMMap revealed that anchors V4 and V5 are additional regions for compound interactions. Compounds forming interactions with the least conserved anchor, V4, may increase 5-LOX selectivity. Inhibitors YS1, YS2 and YS3 produced favorable IC_50_ values of 2.7 µM, 4.2 µM and 5.3 µM, respectively (Fig. 4). Notably, the three identified inhibitors formed interactions with at least anchor V4 or V5 (Fig. 5). Compound YS4 contains a similar structure to the active compounds and was also selected for analysis (Table S1). However, compound YS4 does not form interactions with anchors V4 or V5 and did not show significant 5-LOX inhibition. This suggests a correlation between a compound’s SiMMap score and inhibitory activity (Fig. 4).

In LPS treated mice, inhibiting 5-LOX can dramatically decrease TNF-α and IL-12 and LTB4^35^. Knockdown of 5-LOX expression can inhibit TNF-α-induced IL-6 expression in human synovial fibroblasts^9^. LPS was used as an inflammatory inducer to test whether YS1, YS2 and YS3 would reduce TNF-α expression in a cell-based system. As shown in Fig. 6, we observed a dose-dependent decline of TNF-α protein when treated with the identified inhibitors in RAW 264.7 cells. In addition, IL-6, which can be secreted by macrophages to stimulate the immune response, was decreased (Fig. 6). This suggests that the dramatic decrease of IL-6 observed with the inhibitors YS1, YS2 and YS3 may be due to diminishing TNF-α secretion. Therefore, the in silico screening presented in this study identified 5-LOX inhibitors. Recent studies have suggested that a dual 5-LOX/COX-2 inhibitor may have therapeutic potential^38,39^. However, the focus of this study was on the identification of 5-LOX inhibitors. Further analysis of the identified 5-LOX inhibitors in this study will be needed to determine their effects on COX-2 activity. In total, the identified inhibitors showed a reduction of TNF-α and IL-6 pro-inflammatory mediators by inhibiting 5-LOX in vitro.

5-LOX overexpression is linked to various cancers. This has driven research into 5-LOX targeting inhibitors^14–17^. Identifying novel 5-LOX inhibitor scaffolds may be of great use. We measured the compound similarity between 1,302 known 5-LOX inhibitors and the three inhibitors identified in this study and determined that inhibitors YS1, YS2, and YS3 contain novel structures (Fig. 7). The inhibitors identified in this study contain a maleimide scaffold with an isobutene group attached. They also contain two bulky hydrophobic groups that not only mimics the long-chain of arachidonic acid, but also provide more stable van der Waals interactions within the 5-LOX catalytic site. Together, this presents a novel structure for use as a therapeutic to alleviate 5-LOX mediated inflammation.

Inflammation is present in various diseases, such as asthma, diabetes, and heart disease. Elevated levels of arachidonic acid have been found in sites of inflammation. Consequently, targeting the 5-LOX pathway presents positive therapeutic treatment. Based on our in silico screening results, we identified three novel 5-LOX specific inhibitors with unique scaffolds with the potential for therapeutic benefits. The SiMMap of 5-LOX offers two areas of interest within the catalytic site that can be exploited to increase compound specificity. Further optimization of the compounds will be needed for further study.

## Materials and methods

### Preparations of 5-LOX structure and screening libraries

Structures of 5-LOX were downloaded from Protein Data Bank (PDB), including open-form (PDB code 3V99) and closed-form (PDB code 3O8Y) structures. 5-LOX stays in the closed conformation without the ligand binding. When 5-LOX interacts with arachidonic acid, its catalytic site is induced to open-form structure to accommodate the ligand. The open-form structure contains an incomplete catalytic site. Therefore, we performed homology modeling to construct the open-form structure using MODELLER^40^. The two crystal structures were used as the templates for the modeling. To define the catalytic site, the modeled and closed-form structures were aligned to the open-form structure by a structural alignment method^41^. The bound ligand (i.e., arachidonic acid) was used to identify the catalytic site. Residues that were within 8 Å from arachidonic acid were defined as the catalytic site of 5-LOX.

We collected 118,759 natural compounds from the ZINC compound database^29^ to establish the site-moiety map, including the following seven vendors: AnalytiCon Discovery NP, IBScreen NP, Indofine Natural Products, Molecular Diversity Preservation International, Princeton NP, Selleck BioChemicals NP, and Specs Natural Products. More than 80% of drugs were designed according to the structures of natural compounds^42^. These natural compounds possess a variety of properties for describing physicochemical properties of the catalytic site. Therefore, these natural compounds were used for the establishment of site-moiety map. In addition, a compound library consisting of 525 in-house synthesized compounds was used to identify potential 5-LOX inhibitors with high SiMMap scores.

To validate the SiMMap of 5-LOX, we collected 10 known 5-LOX inhibitors from BindingDB^32^ and 990 compounds randomly selected from ACD, proposed by Bissantz et al*.*^43^. The 10 inhibitors have diverse structures and IC_50_ values of < 1 μM. The 1,000 compounds were docked into the catalytic site and ranked by their docking score or SiMMap score. Then, hit rates were used to assess the performance of the SiMMap-based method.

### Virtual screening and establishment of site-moiety maps

To construct the 5-LOX SiMMap, 118,759 natural compounds were docked using GEMDOCK^30^. The top 2,000 compounds were selected to generate the SiMMap. There are three profiles generated between the compounds and the catalytic site residues and consist of three types: E, H or V. Each profile consists of an *N* × *R* matrix, where *N* and *R* are the number of compounds and interacting residues of 5-LOX, respectively. An interaction profile matrix *M(I)* with type *I* (E, H, or V) is represented as $${M}\left( {I} \right) = \left[ {\begin{array}{*{20}c} {{m}_{1,1} } & \cdots & {{m}_{{1,{R}}} } \\ \vdots & \ddots & \vdots \\ {{m}_{{{N},1}} } & \cdots & {{m}_{{{N},{R}}} } \\ \end{array} } \right]$$ where *m*_*i,r*_ is a binary value for the compound *i* interacting to the residue *r.* Each cell of a matrix is set to 1 or 0 for an interaction or no interaction, respectively. In each profile, consensus interacting residues were identified using a Z-score value. The standard deviation (*σ*) and mean (*μ*) were derived by randomly shuffling the matrix 1,000 times. The Z-score of the residue (*j*) is defined as $Z_{j} = \frac{{f_{j} - \mu }}{\sigma }$, where *f*_*j*_ is the interaction frequency given as $f_{j} = \sum\nolimits_{i = 1}^{N} {\frac{{p_{ij} }}{N}}$*.* A Z-score ≥ 1.645 (95% confidence level) is recorded as statistically significant. These residues were grouped into anchors that contain a preference for one of the three types of interactions mentioned previously. Furthermore, anchors can inform the moiety interactions. The types of moieties were derived from checkmol (https://merian.pch.univie.ac.at/~nhaider/cheminf/cmmm). Together, the identified anchors form the SiMMap of the 5-LOX catalytic site.

We then docked the 525 in-house compounds into the catalytic site of 5-LOX and ranked them based on their SiMMap scores^24^. The SiMMap score of compound *x* was defined as $S(x) = \sum\nolimits_{a = 1}^{n} {AS_{a} (x) + ( - 0.001)\frac{E(x)}{{M^{0.5} }}}$, where *AS*_*a*_(*x*) is the anchor score of compound *x* in anchor *a*, *n* is the anchor number of 5-LOX*, E* (*x*) is the docking score of compound *x*, and *M* is the atom number of compound *x*. *AS*_*a*_(*x*) is set to 1 when compound *x* matches anchor *a*; otherwise it is set to 0. For the EH anchor, *AS*_*a*_(*x*) is set to 0.5 if compound *x* forms hydrogen-bonding interactions with the anchor residues. The term *M*^0.5^ is applied to reduce deleterious effects of selecting compounds with high molecular weight^14^. The compounds were ranked based on their SiMMap score. Three compounds with the highest SiMMap scores and an analogue of the compounds were selected for bioassay. The molecular visualization software PyMol^44^ was used to create the 3D images.

### Molecular dynamic simulation

MD simulations were performed in Discovery Studio^45^ and followed the Standard Dynamics Cascade protocol, which applies a CHARMm forcefield to the protein and the compounds. The simulation was divided into the following five steps: minimization, minimization 2, heating, equilibration and production. Minimization was performed with the steepest descent algorithm, an RMS gradient of 1 and a max steps of 1,000. Next, minimization 2 was performed with the conjugate gradient algorithm, an RMS gradient of 0.1, a max steps of 2000. The system was heated from an initial temperature of 50–300 K with a simulation time of 4 ps and a time step of 2 fs. The equilibration step included a simulation time and time step of 200 ps and 2 fs, respectively. Finally, the production used a simulation time of 10 ns. Other parameters used in the simulation process were set to default values. Final conformations were extracted and interactions were analyzed. The MD simulation models are accessible by the following link: https://bioxgem.life.nctu.edu.tw/5-LOX_MD.zip.

### Evolutionary conservation of residue positions

Conservation scores of residue positions were obtained from the ConSurf server^33^ using the closed-from structure (PDB code 3O8Y) of 5-LOX as the query. The server collected homologous sequences of 5-LOX and generated the multiple sequence alignment. The server then assigned an evolutionary conservation score to each residue position based on the multiple sequence alignment. The evolutionary conservation scores were divided into a discrete scale of nine grades ranging from 1 (variable) to 9 (conserved). The conservation score of an anchor was obtained by averaging grades of the anchor residue positions. In addition, the multiple sequence alignment was used to generate the sequence logos positions using Weblogo^46^.

### Enzyme inhibition assay

An inhibitor screening assay Kit (Cayman Chemical, catalog #760700) was used to validate the potential 5-LOX inhibitors identified by the SiMMap. The compounds were tested in triplicates using the protocol provided by the manufacturer. The assay was carried out by adding the substrate (arachidonic acid), and the potential inhibitors in the concentration range from 1.25 to 40 μM. A known 5-LOX inhibitor, nordihydroguaiaretic acid, was used as the positive control. Inhibitory activity of each compound was measured by detecting the formation of hyperoxides and was calculated by averaging percentage inhibitions observed from the triplicate experiments. IC_50_ values of the inhibitors were generated using non-linear regression function of GraphPad Prism.

### Compound structure analysis of 5-LOX inhibitors

1,302 known 5-LOX inhibitors with IC_50_ values ≤ 10 μM were collected from BindingDB^32^ to compare with YS1, YS2, and YS3. Moiety fingerprints of the inhibitors were generated by checkmol. Each inhibitor contains 90 binary bits. The inhibitor-moiety profile was then clustered using a hierarchical clustering with centroid linkage method. Pearson’s correlation coefficient between fingerprints of two inhibitors was used as the distance to measure the compound similarity.

### Measurement of IL-6 and TNF-expression

The ELISA kits of IL-6 and TNF-α were purchased from BD Biosciences (Hampton, NH, USA). The detection of inflammatory cytokines was followed by instruction manual. Briefly, RAW 264.7 cells were seeded in 96 well-plates (1,000/well) overnight for attachment and cotreated with LPS and compound YS1, YS2 or YS3. LPS stimulated IL-6 and TNF-α release with 100 ng/ml and 10 ng/ml, respectively. The cell culture medium was collected in 1.5 ml eppendorf and centrifuged for 10 min at 1,000 g. The supernatant (samples, 50 μl) was added to each well, which contained 50 μl ELISA Diluent in IL-6 or TNF-α ELISA plate. Incubate the reaction at room temperature for 2 h, and then washed with Wash Buffer for 5 times. Add 100 μl/well of Working Detector, incubate for 1 h at room temperature and then wash. TMB One-Step Substrate Reagent (100 μl/well) was added for 30 min at room temperature in the dark and the reaction was ceased by Stop Solution (50 μl/well). Absorbance at 450 nm was measured to quantify IL-6 or TNF-α amounts.

## Supplementary information

Supplementary file1

Supplementary file2
