# Supplementary material for: A site-moiety map and virtual screening approach for discovery of novel 5-LOX inhibitors
Source: Sci Rep. 2020 Jun 29;10:10510. doi: 10.1038/s41598-020-67420-9 (PMC7324578; doi:10.1038/s41598-020-67420-9)
Supplement: Supplementary file 1 — Supplementary file1 [file 41598_2020_67420_MOESM1_ESM.docx]

**Supplemental Information**

**A Site-Moiety Map and Virtual Screening Approach for Discovery of Novel 5-LOX Inhibitors**

**Kai-Cheng Hsu^1,2,3,4#^, Wei-Chun HuangFu^1,2,3#^, Tony Eight Lin^1,2^, Min-Wu Chao^1^, Tzu-Ying Sung^5^, Yi-Ying Chen^1^, Shiow-Lin Pan^1,2,3,4^, Jih-Chin Lee^6,7^, Shey-Cherng Tzou^8^, Chung-Ming Sun^9^ and Jinn-Moon Yang^5, 8,10*^**

^1^ Graduate Institute of Cancer Biology and Drug Discovery, College of Medical Science and Technology, Taipei Medical University, Taipei, Taiwan

^2^ Ph.D. Program for Cancer Molecular Biology and Drug Discovery, College of Medical Science and Technology, Taipei Medical University, Taipei, Taiwan

^3^ Ph.D. Program in Biotechnology Research and Development, College of Pharmacy, Taipei Medical University

^4^ Biomedical Commercialization Center, Taipei Medical University, Taipei, Taiwan

^5^ Institute of Bioinformatics and Systems Biology, National Chiao Tung University, Hsinchu, Taiwan

^6^ Department of Otolaryngology-Head and Neck Surgery, Tri-Service General Hospital, Taipei, Taiwan

^7^ Department of Otolaryngology-Head and Neck Surgery, National Defense Medical Center, Taipei, Taiwan

^8^ Department of Biological Science and Technology, National Chiao Tung University, Hsinchu, Taiwan

^9^ Department of Applied Chemistry, National Chiao Tung University, Hsinchu, Taiwan

^10^ Center for Intelligent Drug Systems and Smart Bio-devices, National Chiao Tung University, Hsinchu, Taiwan

^*^Corresponding authors.

* E-mail: [moon@faculty.nctu.edu.tw](mailto:moon@faculty.nctu.edu.tw) (JMY)

# These authors contributed equally to this work.


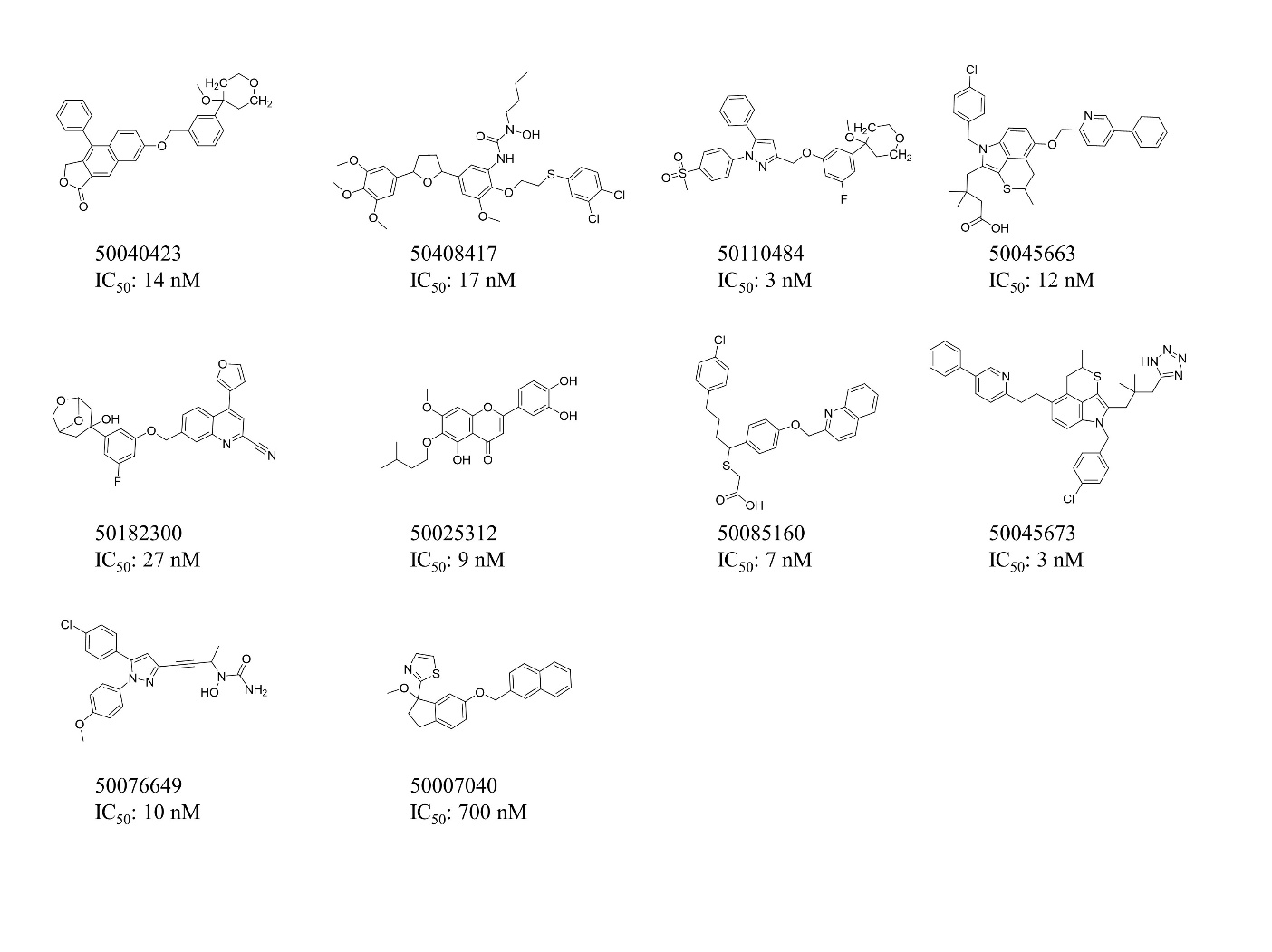


**Supplementary Figure 1.** **Structures of known 5-LOX inhibitors.** The names and structures of the 10 selected 5-LOX inhibitors from BindingDB. Their IC_50_ values are listed as shown.


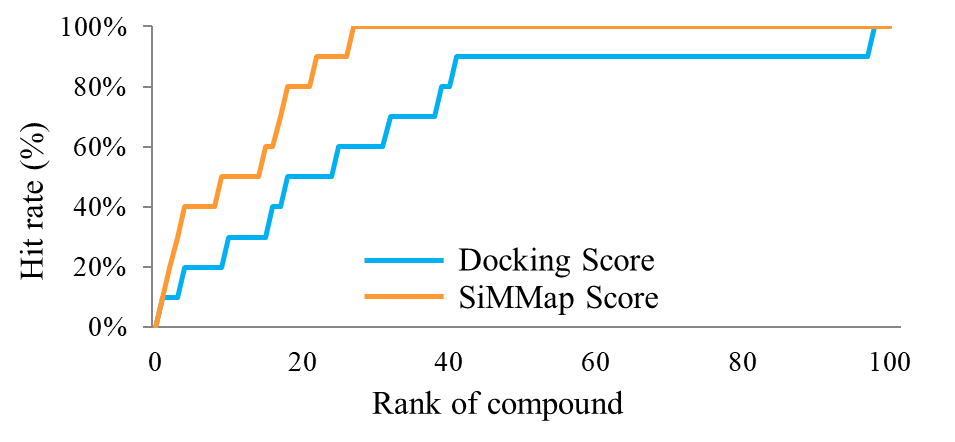


**Supplementary Figure 2.** **Performance of the SiMMap.** 10 known 5-LOX inhibitors were mixed and docked 990 ACD compounds.

Supplementary Figure 3. **Molecular dynamic study of 5-LOX.** The docking pose (purple) and the MD simulation (blue) of compounds (A) YS1, (B) YS2 and (C) YS3. The MD simulation presented favorable binding orientations of the inhibitors in the 5-LOX catalytic site. Red line dictates area with greater hydrophobic interactions. (D) A heatmap presenting the percentage of interactions between the inhibitors and the residues over a 10 nanosecond timeframe.
