# Supplementary figures and images for: A site-moiety map and virtual screening approach for discovery of novel 5-LOX inhibitors
Source: Sci Rep. 2020 Jun 29;10:10510. doi: 10.1038/s41598-020-67420-9 (PMC7324578; doi:10.1038/s41598-020-67420-9)

**Table S1. Structures and ranks of** **top 120 compounds.**

| Molecule | Compound | Rank | Score | EH1 | H1 | V1 | V2 | V3 | V4 | V5 |
| --- | --- | --- | --- | --- | --- | --- | --- | --- | --- | --- |
| 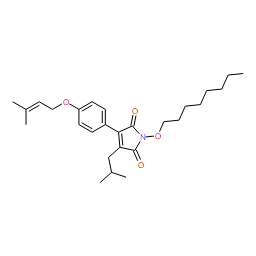 | YS3 | 1 | 5.727 | 0.5 | 1 | 1 | 1 | 1 | 1 | 0 |
| 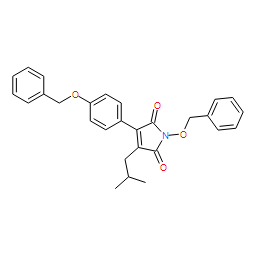 | YS1 | 2 | 5.725 | 0.5 | 1 | 1 | 1 | 0 | 1 | 1 |
| 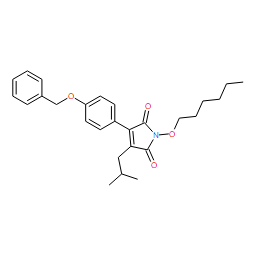 | YS2 | 3 | 5.712 | 0.5 | 1 | 1 | 1 | 1 | 1 | 0 |
| 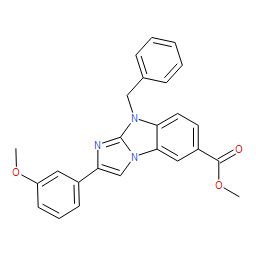 | 16020 | 4 | 5.218 | 0 | 1 | 1 | 1 | 1 | 0 | 1 |
| 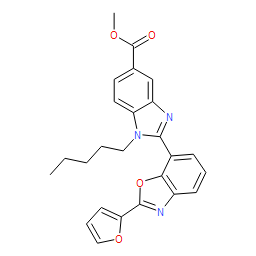 | 26002 | 5 | 5.209 | 0 | 1 | 1 | 1 | 1 | 0 | 1 |
| 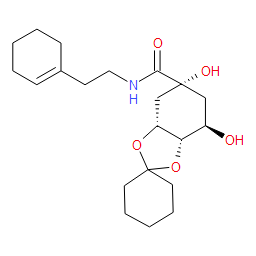 | 17071 | 6 | 5.204 | 0 | 1 | 1 | 1 | 0 | 1 | 1 |
| 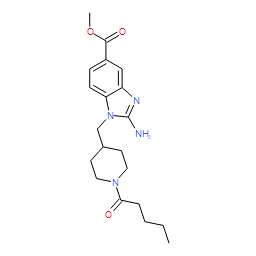 | 17032 | 7 | 5.199 | 0 | 1 | 1 | 1 | 0 | 1 | 1 |
| 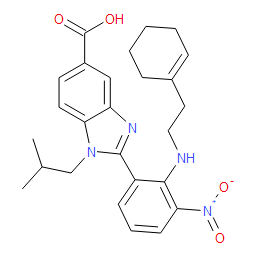 | 16036 | 8 | 5.199 | 0 | 1 | 1 | 1 | 1 | 0 | 1 |
| 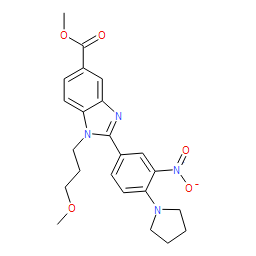 | 12074 | 9 | 5.198 | 0 | 0 | 1 | 1 | 1 | 1 | 1 |
| 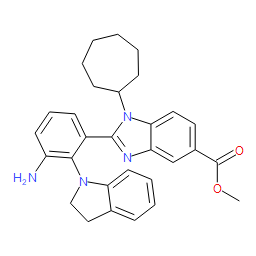 | 17014 | 10 | 5.189 | 0 | 1 | 1 | 1 | 1 | 0 | 1 |
| 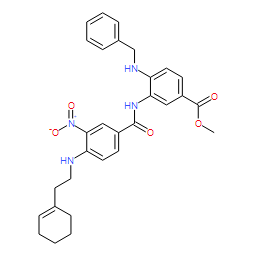 | 15043 | 11 | 5.18 | 0 | 0 | 1 | 1 | 1 | 1 | 1 |
| 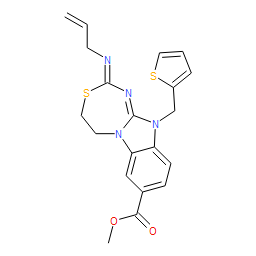 | 17025 | 12 | 5.171 | 0 | 1 | 1 | 1 | 1 | 0 | 1 |
| 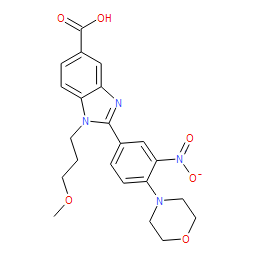 | 12061 | 13 | 4.724 | 0.5 | 1 | 1 | 1 | 1 | 0 | 0 |
| 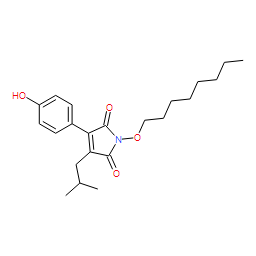 | YS4 | 14 | 4.711 | 0.5 | 1 | 1 | 1 | 1 | 0 | 0 |
| 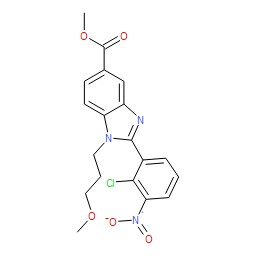 | 17007 | 15 | 4.697 | 0.5 | 1 | 1 | 1 | 1 | 0 | 0 |
| 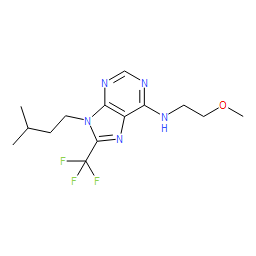 | 26023 | 16 | 4.676 | 0.5 | 1 | 1 | 1 | 0 | 0 | 1 |
| 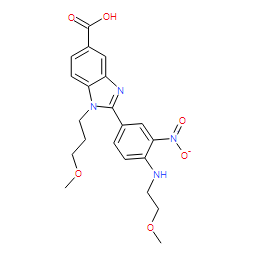 | 12065 | 17 | 4.231 | 1 | 0 | 1 | 1 | 0 | 1 | 0 |
| 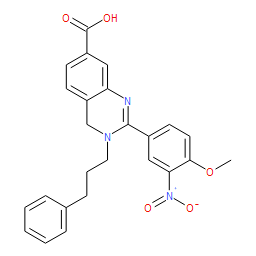 | 12054 | 18 | 4.226 | 0 | 1 | 0 | 1 | 1 | 0 | 1 |
| 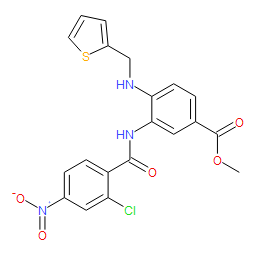 | 17049 | 19 | 4.225 | 0 | 1 | 1 | 1 | 1 | 0 | 0 |
| 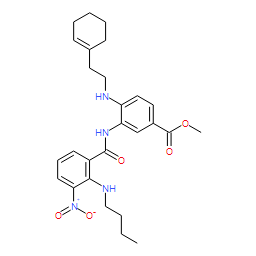 | 16035 | 20 | 4.223 | 0 | 0 | 1 | 1 | 1 | 0 | 1 |
| 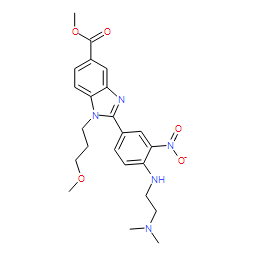 | 12064 | 21 | 4.223 | 0 | 0 | 1 | 1 | 0 | 1 | 1 |
| 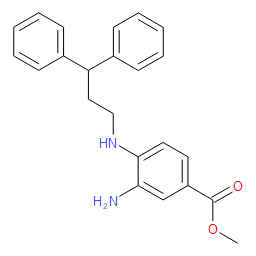 | 16025 | 22 | 4.22 | 0 | 1 | 1 | 1 | 0 | 0 | 1 |
| 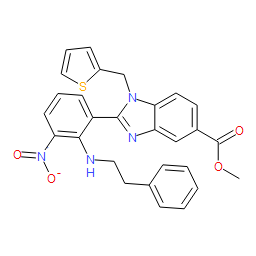 | 18049 | 23 | 4.217 | 0 | 1 | 1 | 1 | 1 | 0 | 0 |
| 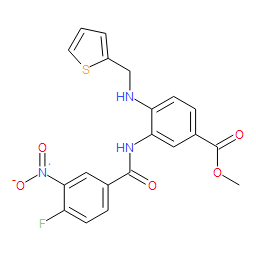 | 18026 | 24 | 4.217 | 0 | 1 | 1 | 1 | 1 | 0 | 0 |
| 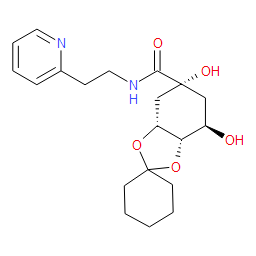 | 17068 | 25 | 4.216 | 0 | 1 | 0 | 1 | 0 | 1 | 1 |
| 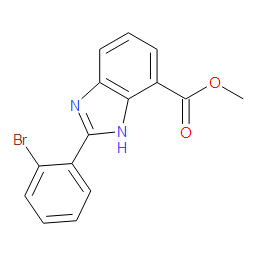 | 18056 | 26 | 4.213 | 0 | 1 | 1 | 1 | 0 | 1 | 0 |
| 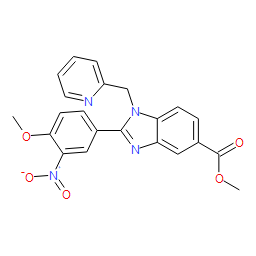 | 13032 | 27 | 4.213 | 0 | 1 | 1 | 1 | 1 | 0 | 0 |
| 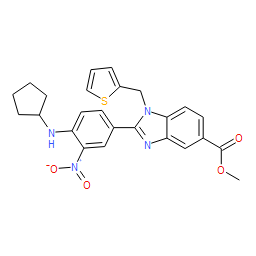 | 18023 | 28 | 4.21 | 0 | 1 | 1 | 1 | 1 | 0 | 0 |
| 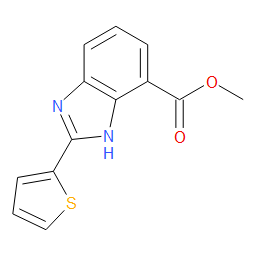 | 18054 | 29 | 4.209 | 0 | 1 | 1 | 1 | 0 | 1 | 0 |
| 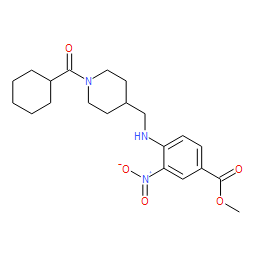 | 28049 | 30 | 4.207 | 0 | 1 | 1 | 1 | 0 | 0 | 1 |
| 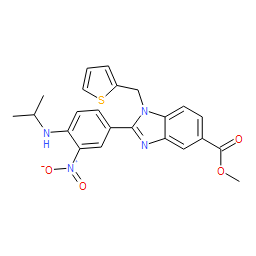 | 18036 | 31 | 4.207 | 0 | 1 | 1 | 1 | 1 | 0 | 0 |
| 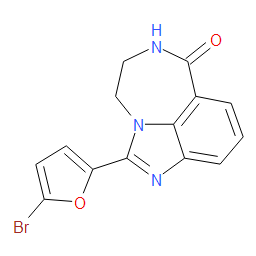 | 27026 | 32 | 4.205 | 0 | 1 | 1 | 1 | 0 | 1 | 0 |
| 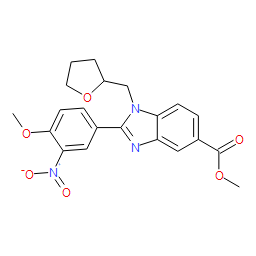 | 13031 | 33 | 4.205 | 0 | 1 | 1 | 1 | 1 | 0 | 0 |
| 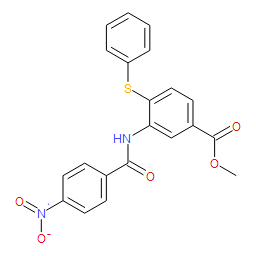 | 13015 | 34 | 4.205 | 0 | 1 | 1 | 1 | 0 | 0 | 1 |
| 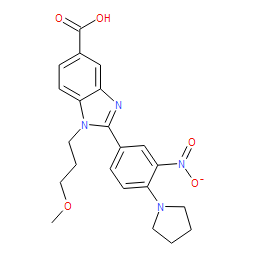 | 12077 | 35 | 4.205 | 0 | 1 | 1 | 1 | 1 | 0 | 0 |
| 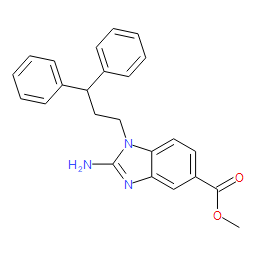 | 16002 | 36 | 4.203 | 0 | 1 | 1 | 1 | 0 | 0 | 1 |
| 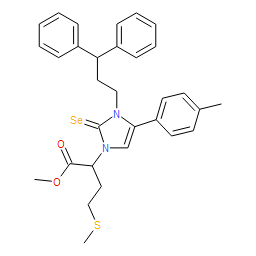 | 15007 | 37 | 4.201 | 0 | 0 | 1 | 1 | 1 | 0 | 1 |
| 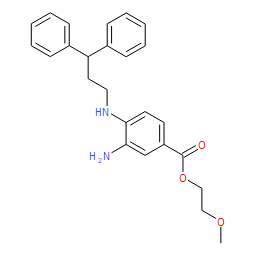 | 28047 | 38 | 4.2 | 0 | 1 | 1 | 1 | 1 | 0 | 0 |
| 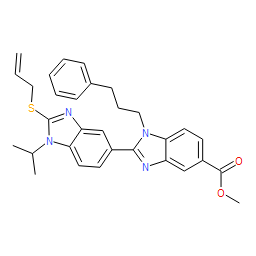 | 17027 | 39 | 4.2 | 0 | 1 | 1 | 1 | 1 | 0 | 0 |
| 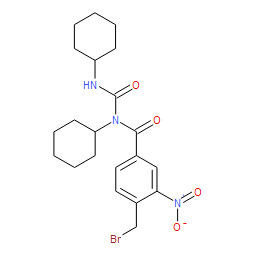 | 13044 | 40 | 4.2 | 0 | 1 | 1 | 1 | 0 | 0 | 1 |
| 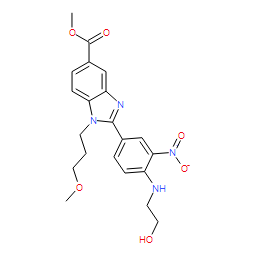 | 12070 | 41 | 4.2 | 0 | 1 | 1 | 1 | 1 | 0 | 0 |
| 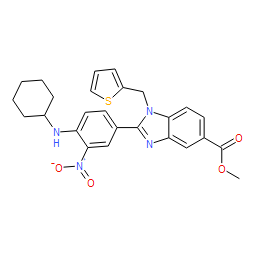 | 18033 | 42 | 4.199 | 0 | 1 | 1 | 1 | 1 | 0 | 0 |
| 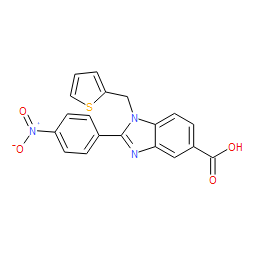 | 17037 | 43 | 4.199 | 0 | 1 | 1 | 1 | 0 | 0 | 1 |
| 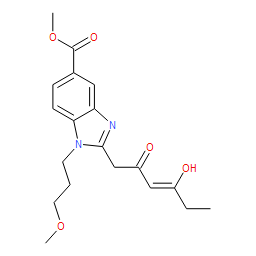 | 26016 | 44 | 4.197 | 0 | 1 | 1 | 1 | 0 | 0 | 1 |
| 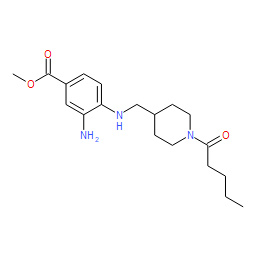 | 17031 | 45 | 4.196 | 0 | 0 | 1 | 1 | 0 | 1 | 1 |
| 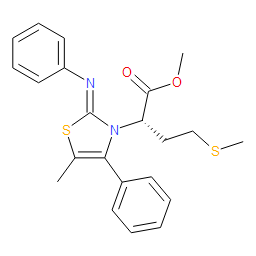 | 15059 | 46 | 4.196 | 0 | 0 | 1 | 1 | 1 | 0 | 1 |
| 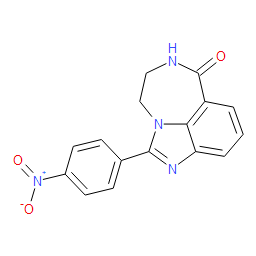 | 27022 | 47 | 4.195 | 0 | 1 | 1 | 1 | 0 | 0 | 1 |
| 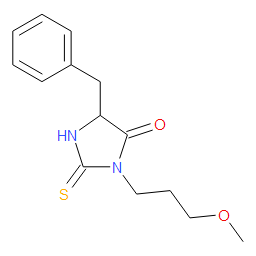 | 16044 | 48 | 4.195 | 0 | 1 | 1 | 1 | 0 | 1 | 0 |
| 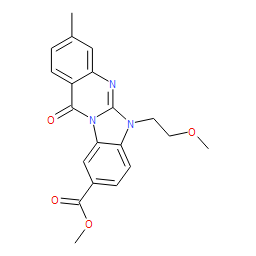 | 17020 | 49 | 4.193 | 0 | 1 | 1 | 1 | 0 | 0 | 1 |
| 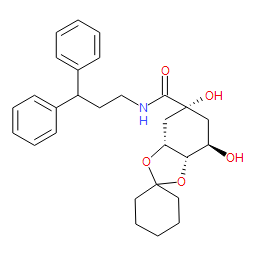 | 17072 | 50 | 4.192 | 0 | 1 | 1 | 1 | 1 | 0 | 0 |
| 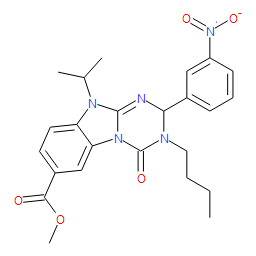 | 13034 | 51 | 4.189 | 0 | 0 | 1 | 1 | 1 | 0 | 1 |
| 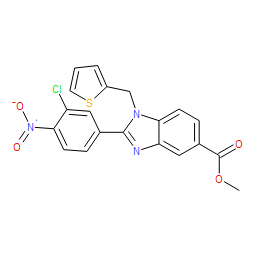 | 18027 | 52 | 4.188 | 0 | 1 | 1 | 1 | 1 | 0 | 0 |
| 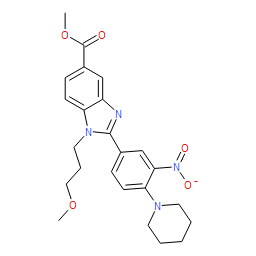 | 12075 | 53 | 4.187 | 0 | 1 | 1 | 1 | 1 | 0 | 0 |
| 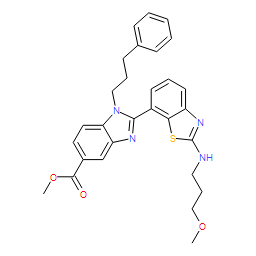 | 26006 | 54 | 4.186 | 0 | 0 | 1 | 1 | 1 | 0 | 1 |
| 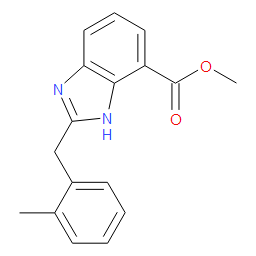 | 18063 | 55 | 4.185 | 0 | 1 | 1 | 1 | 0 | 0 | 1 |
| 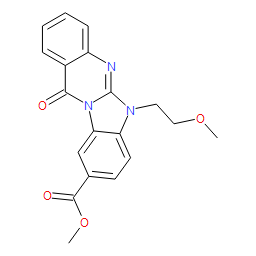 | 17019 | 56 | 4.185 | 0 | 1 | 1 | 1 | 1 | 0 | 0 |
| 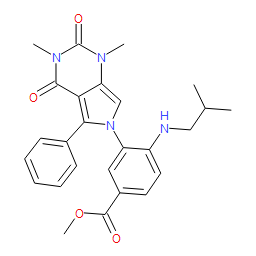 | 22074 | 57 | 4.184 | 0 | 0 | 1 | 1 | 1 | 0 | 1 |
| 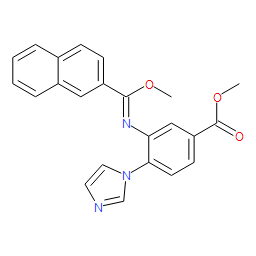 | 17054 | 58 | 4.184 | 0 | 1 | 1 | 1 | 1 | 0 | 0 |
| 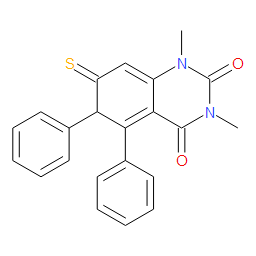 | 26040 | 59 | 4.182 | 0 | 1 | 1 | 1 | 0 | 0 | 1 |
| 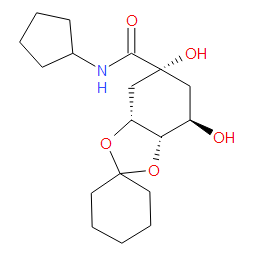 | 17069 | 60 | 4.182 | 0 | 1 | 1 | 1 | 0 | 1 | 0 |
| 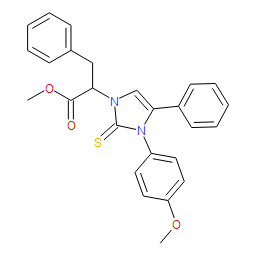 | 15004 | 61 | 4.182 | 0 | 0 | 1 | 1 | 1 | 0 | 1 |
| 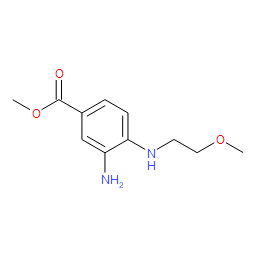 | 27010 | 62 | 4.181 | 0 | 1 | 1 | 1 | 0 | 0 | 1 |
| 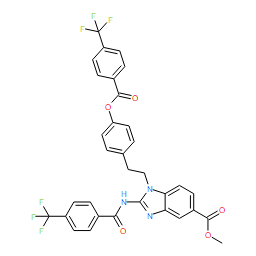 | 12027 | 63 | 4.181 | 0 | 0 | 1 | 1 | 1 | 0 | 1 |
| 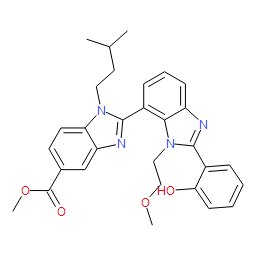 | 16014 | 64 | 4.18 | 0 | 1 | 1 | 1 | 1 | 0 | 0 |
| 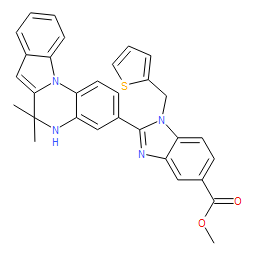 | 13023 | 65 | 4.18 | 0 | 0 | 1 | 1 | 1 | 0 | 1 |
| 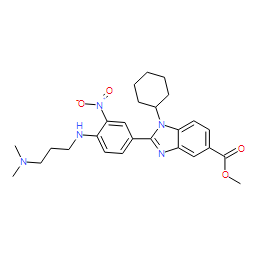 | 28056 | 66 | 4.179 | 0 | 1 | 1 | 1 | 1 | 0 | 0 |
| 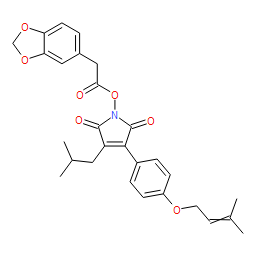 | 15020 | 67 | 4.179 | 0 | 1 | 1 | 1 | 0 | 1 | 0 |
| 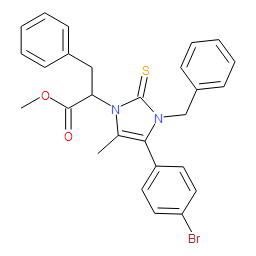 | 15008 | 68 | 4.178 | 0 | 0 | 1 | 1 | 1 | 0 | 1 |
| 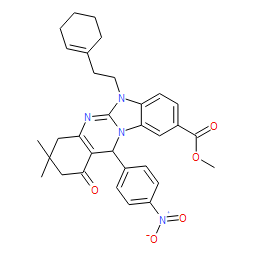 | 13006 | 69 | 4.178 | 0 | 0 | 1 | 1 | 1 | 0 | 1 |
| 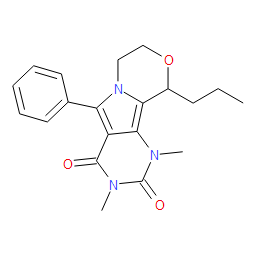 | 27048 | 70 | 4.177 | 0 | 0 | 1 | 1 | 1 | 0 | 1 |
| 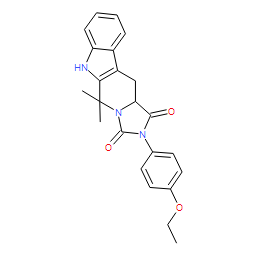 | 13039 | 71 | 4.174 | 0 | 0 | 1 | 1 | 0 | 1 | 1 |
| 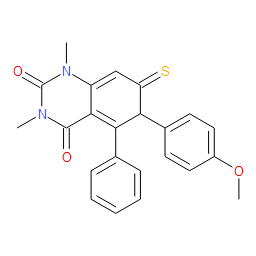 | 26045 | 72 | 4.172 | 0 | 0 | 1 | 1 | 1 | 0 | 1 |
| 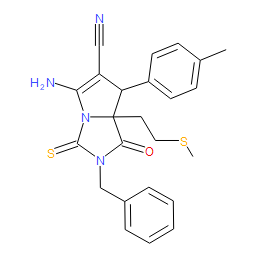 | 15034 | 73 | 4.172 | 0 | 0 | 1 | 1 | 1 | 0 | 1 |
| 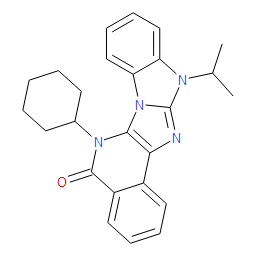 | 12023 | 74 | 4.17 | 0 | 0 | 1 | 1 | 1 | 0 | 1 |
| 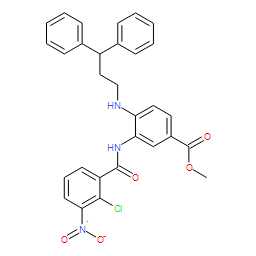 | 15047 | 75 | 4.169 | 0 | 0 | 1 | 1 | 1 | 0 | 1 |
| 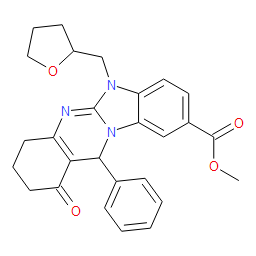 | 13010 | 76 | 4.168 | 0 | 0 | 1 | 1 | 1 | 0 | 1 |
| 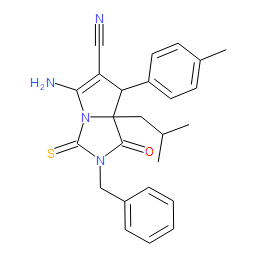 | 15035 | 77 | 4.164 | 0 | 0 | 1 | 1 | 1 | 0 | 1 |
| 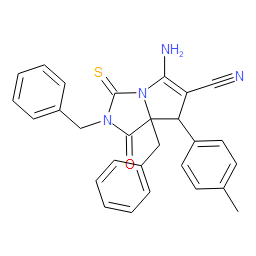 | 15036 | 78 | 4.16 | 0 | 0 | 1 | 1 | 1 | 0 | 1 |
| 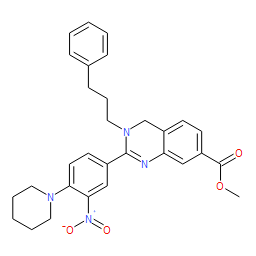 | 12056 | 79 | 4.153 | 0 | 0 | 1 | 1 | 1 | 0 | 1 |
| 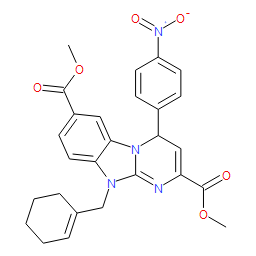 | 12024 | 80 | 4.152 | 0 | 0 | 1 | 1 | 1 | 0 | 1 |
| 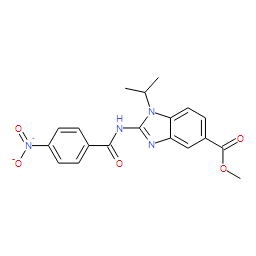 | 28042 | 81 | 3.717 | 0.5 | 1 | 1 | 1 | 0 | 0 | 0 |
| 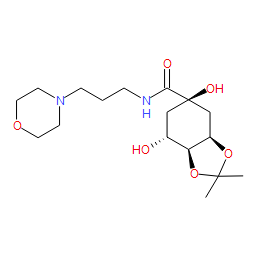 | 17064 | 82 | 3.708 | 0.5 | 1 | 1 | 1 | 0 | 0 | 0 |
| 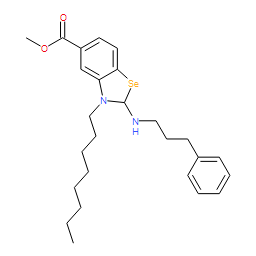 | 26033 | 83 | 3.703 | 0.5 | 0 | 1 | 1 | 0 | 1 | 0 |
| 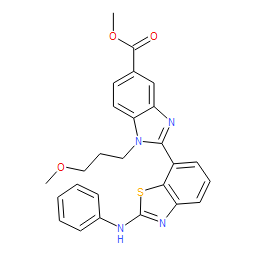 | 26007 | 84 | 3.694 | 0.5 | 0 | 1 | 1 | 1 | 0 | 0 |
| 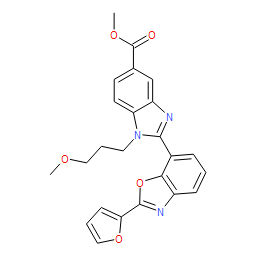 | 26004 | 85 | 3.692 | 0.5 | 0 | 0 | 1 | 1 | 0 | 1 |
| 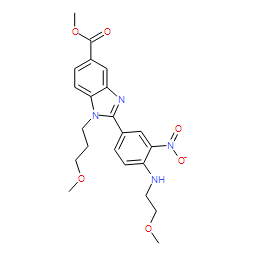 | 12063 | 86 | 3.69 | 0.5 | 0 | 1 | 1 | 0 | 1 | 0 |
| 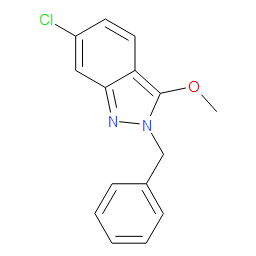 | 13008 | 87 | 3.686 | 0.5 | 0 | 1 | 1 | 0 | 0 | 1 |
| 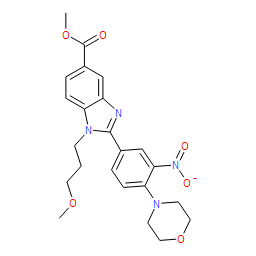 | 12068 | 88 | 3.686 | 0.5 | 0 | 1 | 1 | 1 | 0 | 0 |
| 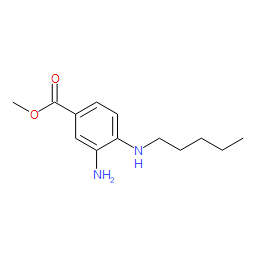 | 26025 | 89 | 3.685 | 0.5 | 0 | 1 | 1 | 0 | 0 | 1 |
| 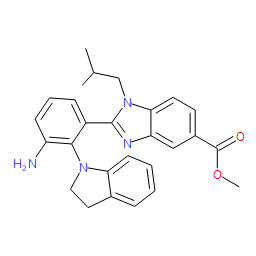 | 17004 | 90 | 3.678 | 0.5 | 0 | 1 | 0 | 1 | 0 | 1 |
| 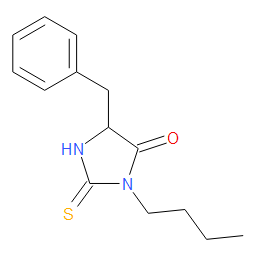 | 16046 | 91 | 3.676 | 0.5 | 0 | 1 | 1 | 0 | 0 | 1 |
| 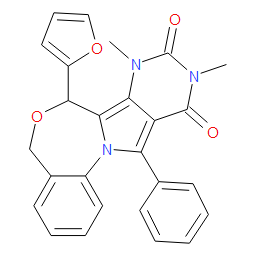 | 27040 | 92 | 3.666 | 0.5 | 0 | 1 | 1 | 1 | 0 | 0 |
| 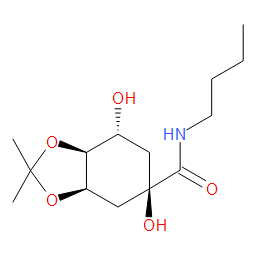 | 17060 | 93 | 3.665 | 0.5 | 0 | 1 | 1 | 0 | 0 | 1 |
| 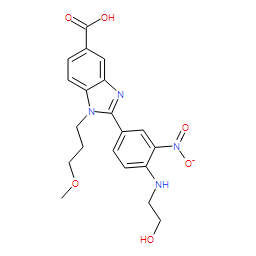 | 12067 | 94 | 3.241 | 1 | 0 | 1 | 1 | 0 | 0 | 0 |
| 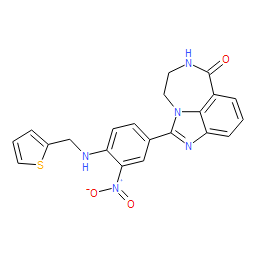 | 27051 | 95 | 3.225 | 0 | 0 | 1 | 1 | 0 | 0 | 1 |
| 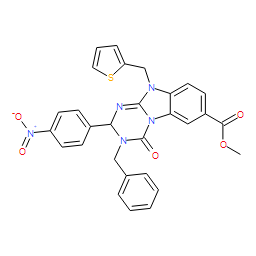 | 13035 | 96 | 3.219 | 0 | 0 | 1 | 1 | 1 | 0 | 0 |
| 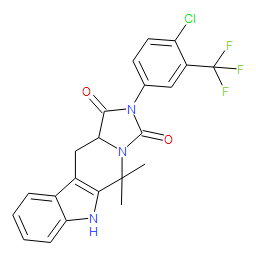 | 13043 | 97 | 3.216 | 0 | 0 | 1 | 1 | 0 | 1 | 0 |
| 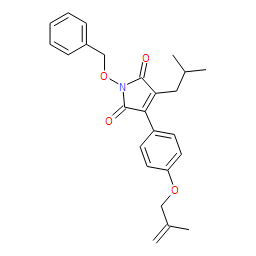 | 15041 | 98 | 3.215 | 0 | 0 | 1 | 1 | 1 | 0 | 0 |
| 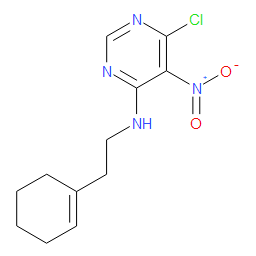 | 26024 | 99 | 3.212 | 0 | 1 | 1 | 1 | 0 | 0 | 0 |
| 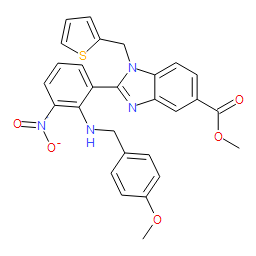 | 18051 | 100 | 3.212 | 0 | 1 | 0 | 1 | 0 | 1 | 0 |
|  | 17043 | 101 | 3.212 | 0 | 0 | 1 | 1 | 0 | 1 | 0 |
|  | 12076 | 102 | 3.212 | 0 | 0 | 1 | 1 | 0 | 0 | 1 |
|  | 26056 | 103 | 3.21 | 0 | 0 | 1 | 1 | 1 | 0 | 0 |
|  | 12042 | 104 | 3.21 | 0 | 1 | 1 | 1 | 0 | 0 | 0 |
|  | 12046 | 105 | 3.208 | 0 | 0 | 1 | 1 | 1 | 0 | 0 |
|  | 17044 | 106 | 3.207 | 0 | 0 | 1 | 1 | 0 | 0 | 1 |
|  | 17041 | 107 | 3.207 | 0 | 0 | 1 | 1 | 0 | 0 | 1 |
|  | 12037 | 108 | 3.203 | 0 | 1 | 1 | 1 | 0 | 0 | 0 |
|  | 15057 | 109 | 3.202 | 0 | 0 | 1 | 1 | 0 | 1 | 0 |
|  | 27029 | 110 | 3.2 | 0 | 0 | 1 | 1 | 0 | 1 | 0 |
|  | 26014 | 111 | 3.2 | 0 | 1 | 1 | 1 | 0 | 0 | 0 |
|  | 12044 | 112 | 3.2 | 0 | 0 | 1 | 1 | 0 | 0 | 1 |
|  | 12035 | 113 | 3.2 | 0 | 0 | 1 | 1 | 0 | 0 | 1 |
|  | 17030 | 114 | 3.199 | 0 | 0 | 1 | 1 | 0 | 1 | 0 |
|  | 17029 | 115 | 3.199 | 0 | 0 | 1 | 1 | 0 | 1 | 0 |
|  | 13024 | 116 | 3.199 | 0 | 0 | 1 | 0 | 1 | 0 | 1 |
|  | 17023 | 117 | 3.198 | 0 | 0 | 1 | 1 | 1 | 0 | 0 |
|  | 15029 | 118 | 3.198 | 0 | 0 | 1 | 1 | 1 | 0 | 0 |
|  | 13051 | 119 | 3.198 | 0 | 1 | 1 | 1 | 0 | 0 | 0 |
|  | 12030 | 120 | 3.197 | 0 | 0 | 1 | 1 | 0 | 0 | 1 |

Supplement: Supplementary file 2 — Supplementary file2 [file 41598_2020_67420_MOESM2_ESM.docx]
